# Considerations for the Use of Phage Therapy in Clinical Practice

**DOI:** 10.1128/aac.02071-21

**Published:** 2022-03-15

**Authors:** Gina A. Suh, Thomas P. Lodise, Pranita D. Tamma, Jane M. Knisely, Jose Alexander, Saima Aslam, Karen D. Barton, Erica Bizzell, Katherine M. C. Totten, Joseph L. Campbell, Benjamin K. Chan, Scott A. Cunningham, Katherine E. Goodman, Kerryl E. Greenwood-Quaintance, Anthony D. Harris, Shayla Hesse, Anthony Maresso, Veronique Nussenblatt, David Pride, Michael J. Rybak, Zoe Sund, David van Duin, Daria Van Tyne, Robin Patel, for the Antibacterial Resistance Leadership Group

**Affiliations:** a Mayo Clinic, Rochester, Minnesota, USA; b Albany College of Pharmacy and Health Sciences, Albany, New York, USA; c Johns Hopkins University School of Medicine, Baltimore, Maryland, USA; d National Institutes of Health, Bethesda, Maryland, USA; e Advent Health, Orlando, Florida, USA; f University of California San Diego, San Diego, California, USA; g Duke University, Durham, North Carolina, USA; h Yale University, New Haven, Connecticut, USA; i University of Maryland School of Medicine, Baltimore, Maryland, USA; j Baylor College of Medicine, Houston, Texas, USA; k College of Pharmacy & Health Sciences and School of Medicine, Wayne State University, Detroit, Michigan, USA; l University of North Carolina at Chapel Hill, Chapel Hill, North Carolina, USA; m University of Pittsburgh School of Medicine, Pittsburgh, Pennsylvania, USA

**Keywords:** *Staphylococcus aureus*, *Pseudomonas aeruginosa*, biofilms, phages

## Abstract

Increasing antimicrobial resistance and medical device-related infections have led to a renewed interest in phage therapy as an alternative or adjunct to conventional antimicrobials. Expanded access and compassionate use cases have risen exponentially but have varied widely in approach, methodology, and clinical situations in which phage therapy might be considered. Large gaps in knowledge contribute to heterogeneity in approach and lack of consensus in many important clinical areas. The Antibacterial Resistance Leadership Group (ARLG) has convened a panel of experts in phage therapy, clinical microbiology, infectious diseases, and pharmacology, who worked with regulatory experts and a funding agency to identify questions based on a clinical framework and divided them into three themes: potential clinical situations in which phage therapy might be considered, laboratory testing, and pharmacokinetic considerations. Suggestions are provided as answers to a series of questions intended to inform clinicians considering experimental phage therapy for patients in their clinical practices.

## INTRODUCTION

Phage (bacteriophage) therapy has gained resurgent interest in recent years due to the lack of therapeutic options for patients unresponsive to conventional antimicrobials. Antimicrobial resistance and increasing use of implantable devices, which are prone to biofilm-mediated infections, have contributed to decreased effectiveness of antibiotics. Patients with infected medical devices are sometimes unable to undergo surgical source control due to dependence on a life-sustaining device, as in the case of left ventricular assist device infections, or due to devastating consequences of removal of devices on functional capacity, as in the case of periprosthetic joint infection (PJI). Such patients may be left with a chronically infected indwelling device. Other infections, such as urinary tract infections (UTIs), respiratory infections in patients with chronic lung disease, or skin and skin structure infections, may recur despite treatment with appropriate antibiotics. As such, clinicians are increasingly seeking new options for their patients with infections refractory to antibiotic therapy, one of which is phage therapy.

In late 2020, the National Institute of Allergy and Infectious Diseases (NIAID) and the Antibacterial Resistance Leadership Group (ARLG), funded by NIAID, convened a task force comprised of experts in the field of phage therapy, clinical microbiology, antimicrobial resistance, and pharmacology who worked with regulatory experts and a funding agency to develop a series of questions addressing issues surrounding experimental use of phage therapy in clinical practice. These recommendations are not the positions of the National Institutes of Health (NIH) or NIAID; however, several NIAID scientists with expertise in phage therapy contributed to this work. A review of the literature was conducted and each question answered, with gaps in knowledge identified, where applicable, by consensus of ARLG Phage Taskforce members. The ESKAPE (Enterococcus faecium, Staphylococcus aureus, Klebsiella pneumoniae, Acinetobacter baumannii, Pseudomonas aeruginosa, and Enterobacter species) bacteria alongside resistant Gram-positive bacteria were prioritized to maintain alignment with the primary mission of ARLG. The ARLG Phage Taskforce was divided into three subgroups, clinical, laboratory testing, and pharmacokinetic subgroups, with representation from NIAID staff on each; each subgroup met regularly to identify relevant questions and examine the associated literature. This report is the product of these efforts, offered as a resource to familiarize clinicians with issues surrounding clinical use of phage therapy and provide an evidence-based evaluation of circumstances where this experimental therapy might be considered, acknowledging that at this point, no recommendation can be made to support routine clinical use of phage therapy under any circumstance.

## METHODOLOGY

Each subgroup of the ARLG Phage Taskforce came up with clinically relevant questions surrounding phage therapy. Questions were answered by performing a literature review. A professional medical librarian conducted an electronic search of the literature. Medline (Ovid), Embase (Elsevier), and Cochrane Central Registry of Controlled Trials (Wiley) were searched to identify studies investigating phage therapy from a clinical standpoint. The search was limited to years 2000 to 2020 and included only English language studies; editorials, comments, letters, and conference abstracts were excluded. The search returned 14,841 abstracts, which were screened by taskforce members, yielding 968 manuscripts that were reviewed for the document. Important questions, themes, and gaps in knowledge were identified and divided into three sections, clinical situations in which phage therapy might be considered, laboratory testing, and pharmacokinetics, each of which was handled by separate subgroups. Each subgroup met regularly via virtual meetings, and a consensus was reached on suggestions. Given gaps in knowledge and scarcity of data in the field, this document is intended to provide considerations to clinicians considering use of experimental phage therapy based on extensive literature review, clinical experience, and expert opinion. The literature search was subsequently updated to include papers published as of 9 August 2021.

## QUESTION 1: FOR WHICH INFECTIONS CAN PHAGE THERAPY BE CONSIDERED?

### Suggestion.

The ARLG Phage Taskforce suggests that experimental phage therapy can be considered for a variety of infections refractory to conventional antibiotics, including respiratory tract infections, infections involving devices that cannot be removed, osteoarticular infections, UTIs, gastrointestinal infections, endovascular infections, and other source infections. Bacteriophage therapy is a consideration for bacterial but not fungal, viral, or parasitic infection.

### Rationale.

Phage therapy is an investigational anti-infective treatment for refractory, multidrug-resistant (MDR), and/or biofilm-mediated infections. These situations include chronic and recurrent infections such as UTIs, rhinosinusitis, skin and soft-tissue infections, and MDR respiratory infections, as described below. Biofilm-associated infections include PJI, osteomyelitis involving hardware, cardiac device infection, and respiratory infections in the setting of cystic fibrosis (CF). In general, phage therapy should be limited to treatment of infections after intolerance or ineffectiveness of antibiotic therapy has been demonstrated. These scenarios generally occur in the setting of MDR infections and/or hardware-associated infections not amenable to source control. Phages with activity against Staphylococcus aureus and Pseudomonas aeruginosa are most readily available from academic as well as some commercial entities. Phages with activity against organisms such as Klebsiella pneumoniae, Enterococcus faecalis and *faecium*, Acinetobacter baumannii, Escherichia coli, Proteus mirabilis, *Burkholderia* species, and nontuberculous mycobacteria, among others, are less readily available but have been used to treat human infections. Phage therapy is currently limited to treatment of bacterial infections; however, data are emerging to indicate that nonbacterial organisms such as Aspergillus species can be treated in the future; this is not the subject of this work (1, 2). Members of the ARLG Phage Taskforce reviewed available data on experience with phage therapy for treatment of specific infection types. Table 1 summarizes published clinical experiences from case reports and case series on the use of phage therapy to treat human infections in recent years. Gleaning the potential benefit of phage therapy from the published literature is challenging for several reasons. First, most clinical publications do not include a control group. Second, there is heterogeneity in the route of administration, dose of phage, number of phages (i.e., monotherapy versus cocktail), and duration of phage therapy administered across studies. Third, phages are almost universally administered in addition to antibiotics, making an understanding of the specific role of phages in impacting clinical outcomes difficult to discern. Fourth, the use of phage susceptibility testing (PST) to ensure the phages administered are indeed active against the bacterial pathogen has been inconsistent and is nonstandardized (see Question 7). Finally, publication bias exists, with reports of unfavorable outcomes being less likely to be published than those with favorable outcomes. Below we summarize some of the most common infection types for which phage therapy has been used to date.

**TABLE 1:** Summary of recent published clinical reports and case series of phage therapy in humans^*a*^

Article (reference); PMID	Organism	Demographics	Infectious syndrome	Route of phage administration	No. of phage administered	Highest dose of phage administered (PFU)	Frequency of phage administration	Duration of phage administration	Other anti-infectives administered simultaneously	Clinical outcomes	Survived initial infection	Adverse events	Follow-up period
Corbellino, 2020 (5); 31414123	Klebsiella pneumoniae	57-yr-old female	Urinary tract infection (from intestinal colonization)	Oral and intrarectal	1 phage	1 × 10^6^	q12h	3 wk	Yes	Remained free of infection with the colonization strain	Yes	No	11 mo
Aslam et al., 2020 (7); 33005701	Staphylococcus aureus	61-yr-old male	Periprosthetic joint infection	i.v. and direct application	3 phages	3 × 10^9^	i.v. q12h; intra-articular injection once	2 wk	Yes	Clinical improvement at 2 wk but recurrence on day 17 that necessitated additional antibiotics, phage therapy, and surgery	Yes	None reported	7 mo
Aslam et al., 2020 (7); 33005701	Escherichia coli	56-yr-old male	Urinary tract infection	i.v.	4 phages	1 × 10^9^	q12h	2 wk	Yes	No clinical symptoms for 12 wk but urine culture remained positive	Yes	None reported	12 wk
Aslam et al., 2020 (7); 33005701	Pseudomonas aeruginosa	64-yr-old male	Aortic graft infection		3 phages	2.6 × 10^6^	q12h	6 wk	Yes	Clinical resolution	Yes	None reported	12 wk
Kuipers et al., 2019 (8); 31611357	K. pneumoniae	58-yr old male	Urinary tract infection	Oral and direct application	Unknown	Unknown	q12–24h	16 wk	Yes	Symptomatic relief	Yes	None reported	40 wk
Bao et al., 2020 (9); 32212918	K. pneumoniae	63-yr-old female	Urinary tract infection	Direct application	5-6 phage cocktails	5 × 10^8^	q24h	5 days	Yes	Clinical cure	Yes	None reported	6 mo
Khawaldeh et al., 2011 (10); 21737541	P. aeruginosa	67-yr-old female	Urinary tract infection	Direct application	6 phages	6 × 10^6^	q12h	10 days	Yes	Symptomatic relief; microbiological improvement with nearly 2-log reduction in P. aeruginosa urinary concentrations	Yes	None reported	12 mo
Aslam et al., 2020 (7); 33005701	P. aeruginosa	82-yr-old male	Ventricular assist device infection	i.v. and direct application	4 phages	7.58 × 105	q8–12h	∼10 wk	Yes	Recurrent bacteremia within 1 wk of completing phage therapy; retreated with phage with recurrent bacteremia 4 wk into second episode while receiving phage	Yes	Fever, wheezing, shortness of breath after two infusions of 10^11^ PFU/mL concn; same phage well tolerated at 10^10^PFU/mL	5 mo
Aslam et al., 2020 (7); 33005701	P. aeruginosa	60-yr-old male	Ventricular assist device infection	i.v.	3 phages	1.9 × 10^7^	q8h	6 wk	Yes	Developed bacteremia 1 wk after starting phage therapy; developed recurrent purulent drainage following end of phage therapy	Yes	None reported	6 wk
Jikia et al., 2005 (17); 15663496	S. aureus	52-yr-old male	Skin and soft tissue infection	Direct application	≥5 phages	Not described	Once	Once	Yes	Clinical recovery within 7 days	Yes	None reported	7 days
Law et al., 2019 (20); 31102236	P. aeruginosa	26-yr-old female	Cystic fibrosis exacerbation	i.v.	4 phages	4 × 10^9^	q6h	8 wk	yes	Clinical improvement 7 days into therapy; no CF exacerbations within 100 days following end of phage therapy	Yes	None reported	100 days
Gainey et al., 2020 (21); 32662948	Achromobacter species	10-yr-old female	Cystic fibrosis exacerbation in a cystic fibrosis patient	i.v.	1 phage	Not described	q24h	28 days (14 days without antibiotics and 14 days with antibiotics)	Yes	FEV_1_ improved when administered with antibiotics; *Achromobacter* sp. could not be recovered up to 16 wk after treatment	Yes	None reported	16 wk
Hoyle et al., 2018 (22); 29777836	Achromobacter xylosoxidans	17-yr-old female	Cystic fibrosis exacerbation in a cystic fibrosis patient	Oral and nebulized	2 phages	3 × 10^8^	q24h	20 days	Yes	Dyspnea resolved and cough decreased; FEV_1_ increased from 54% to 84%	Yes	None reported	12 mo
Dedrick et al., 2019 (23); 31068712	Mycobacterium abscessus	15-yr-old female	Pneumonia in a lung transplant recipient with disseminated lesions	i.v.	3 phages	1 × 10^9^	q12h	∼4 mo	Yes	Clinical improvement, including resolution of infected skin nodules	Yes	Sweating and flushing for 2 day	6 mo
Aslam et al., 2019 (24); 31207123	P. aeruginosa	67-yr-old male	Pneumonia in a lung transplant recipient	i.v. and nebulized	3-5 phages	5 × 10^9^	i.v. q2–6h and nebulized q6–12h	29 days	Yes	New hospitalization for pneumonia on day 46 (with P. aeruginosa); recovered well from both episodes of pneumonia	Yes	None reported	29 days
Aslam et al., 2019 (24); 31207123	Burkholderia dolosa	28-yr-old male	Pneumonia in a lung transplant recipient	i.v.	1 phage	3.5 × 10^7^	q12–24h	12 wk	Yes	Recovered	No	None reported	Died at 6 mo from multiorgan system failure
Aslam et al., 2019 (24); 31207123	P. aeruginosa	52-yr-old female	Pneumonia in a lung transplant recipient	i.v.	4 phages	4 × 10^9^	q12h	4 wk	Yes	Recovered	Yes	None reported	60 days
Dedrick et al., 2021 (25); 34239133	M. abscessus	81-yr-old male	Refractory pulmonary infection in the setting of bronchiectasis	i.v.	3 phages	1 × 10^9^	q12h	6 mo	Yes	Initial treatment response, followed by increased bacterial counts and ultimately treatment failure	Yes	None	NA
Wu et al., 2021 (26); 33703996	A. baumannii	62-yr-old male	Secondary pneumonia in COVID-19	Nebulized	1 phage, then 2 phages	1 × 10^9^	2 administrations, 1 day apart	2 days	Yes	Decline in semiquantitative *A. baumannii* burden; clinical cure	Yes	Fever, IL-6, IL-8 cytokine storm; resolved after 1 day	30 days
Wu et al., 2021 (26); 33703996	A. baumannii	64-yr-old male	Secondary pneumonia in COVID-19	Nebulized	2 phages	1 × 10^9^	2 administrations, 1 hour apart	1 day	Yes	Decline in semiquantitative *A. baumannii* burden; clinical cure	Yes	None reported	9 days
Wu et al., 2021 (26); 33703996	A. baumannii	81-yr-old male	Secondary pneumonia in COVID-19	Nebulized	2 phages	1 × 10^9^	2 administrations, 1 hour apart	1 day	Yes	Decline in semiquantitative *A. baumannii* burden; died day 10	No	None reported	10 days
Wu et al., 2021 (26); 33703996	A. baumannii	78-yr-old male	Secondary pneumonia in COVID-19	Nebulized	2 phages	1 × 10^9^	2 administrations, 1 hour apart	1 day	Yes	Decline in semiquantitative *A. baumannii* burden; died day 40 of K. pneumoniae infection	No	None reported	40 days
Maddocks et al., 2019 (27); 31437402	P. aeruginosa	77-yr-old female	Pneumonia and empyema	Nebulized and i.v.	4 phages	1 × 10^9^	q12h	7 days	Yes	Clinical cure	Yes	None reported	6 mo
Nir-Paz et al., 2019 (32); 30869755	A. baumannii, K. pneumoniae	42-yr-old male	Osteomyelitis	i.v.	2 phages	5 × 10^7^	q8h	11 days	Yes	Rapid tissue healing within a few days; wound healed by 2 wk; prevented likely leg amputation	Yes	None reported	8 mo
LaVergne et al., 2018 (33); 29687015	A. baumannii	77-yr-old male	Craniotomy site infection	i.v.	1 phage	2.14 × 10^7^	q2h	8 days	Yes	Direct improvement of craniotomy site	No	2 h after first dose became transiently hy potensive (no pressors)	Died at day 20 from withdrawal of care due to poor neurologic status
Cano et al., 2020 (34); 32699879	K. pneumoniae	62-yr-old male	Periprosthetic joint infection	i.v.	1 phage	6.3 × 10^10^	q24h	40 days	Yes	Reduction in biofilm mass within 24 h; improved mobility	Yes	None reported	8 mo
Khatami et al., 2021 (35); 34369652	P. aeruginosa	7-yr-old female	Septic arthritis and osteomyelitis	i.v.	1 phage	1 × 10^11^	q12–q24	2 wk	Yes	Clinical recovery	Yes	Fever, transient increase in heel pain	5 mo
Onsea et al., 2019 (36); 31548497	P. aeruginosa, Staphylococcus epidermidis	Unknown	Osteomyelitis of femur	Direct application	2 phages	2 × 10^7^	q8h	10 days	Yes	Recurrence of infection 8 mo later	Yes	None reported	16 mo
Onsea et al., 2019 (36); 31548497	*Enterococcus faecalis*	Unknown	Osteomyelitis of femur	Direct application	6 phages	Unknown	q8h	7 days	Yes	Recovered	Yes	None reported	8 mo
Onsea et al., 2019 (36); 31548497	P. aeruginosa, S. epidermidis	Unknown	Osteomyelitis of pelvis	Direct application	2 phages	2 × 10^7^	q8h	7 days	Yes	Recovered	Yes	Direct redness, pain	16 mo
Onsea et al., 2019 (36); 31548497	Streptococcus agalactiae, S. aureus	Unknown	Osteomyelitis of femur	Direct application	2 phages	2 × 10^7^	q8h	9 days	Yes	Recovered	Yes	None reported	8 mo
Ferry et al., 2020 (37); 32850878	S. aureus	49-yr-old male	Periprosthetic joint infection	Direct application	2 phages	2 × 10^10^	Once	1 day	Yes	Recurrence of infection with different pathogens leading eventually to amputation	Yes	None reported	12 mo
Ferry et al., 2018 (38); 30474047	S. aureus, P. aeruginosa (suspected but not proven)	80-yr-old female	Periprosthetic joint infection	Direct application	6 phages	6 × 10^10^	Once	1 day	Yes	Favorable outcome	Yes	None reported	18 mo
Ferry et al., 2018 (39); 30060002	P. aeruginosa	60-yr-old male	Osteomyelitis	Direct application	4 phages	(1.2–9.7) × 10^8^	Every 3 days for 4 h at a time	12 days	Yes	Gross appearance of wound improved by day 14	No	None reported	Died at day 45 due to spine metastases
Tkhilaishvili et al., 2019 (40); 31527029	P. aeruginosa	80-yr-old female	Periprosthetic joint infection	Direct application	1 phage	1 × 10^8^	q8h	5 days	Yes	No P. aeruginosa isolated in joint fluid samples in first 5 days and 4 wk after phage instillation and up to 10 mo later	Yes	None reported	10 mo
Ferry et al., 2021 (41); 34026768	P. aeruginosa	88-yr-old male	Periprosthetic joint infection	Direct application	2 phages	3 × 10^10^	Once	Once	Yes	Resolution	Yes	None reported	1 yr
Ferry et al., 2020 (42); 33304911	S. aureus	80-yr-old male	Periprosthetic joint infection	Direct application	3 phages	1 × 10^9^	Once	Once	Yes	Resolution	Yes	None reported	2 yr
Ferry et al., 2020 (42); 33304911	S. aureus (suspected but not proven)	84-yr-old male	Periprosthetic joint infection	Direct application	3 phages	1 × 10^9^	Once	Once	Yes	Resolution	Yes	None reported	7 mo
Doub et al., 2021 (43); 33800146	S. epidermidis	79-yr-old female	Periprosthetic joint infection	Direct application	1 phage	2 × 10^10^	Once	Once	Yes	No recurrence on chronic suppresion; resolution of chronic anemia	Yes	Trans ient elevation in liver enzymes	5 mo
Ferry et al., 2020 (43); 33304911	S. aureus	83-yr-old female	Periprosthetic joint infection	Direct application	3 phages	1 × 10^9^	Once	Once	Yes	Pain-free and functional joint but with intermittent drainage from fistula	Yes	None reported	11 mo
Ramirez-Sanchez et al., 2021 (44); 34205687	S. aureus	61-yr-old female	Periprosthetic joint infection	i.v. and direct application	1 phage	3 × 10^10^	q12h	6 wk	Yes	Clinical cure	Yes	None reported	20 mo
Doub et al., 2020 (45); 32397354	S. aureus	72-yr-old male	Periprosthetic joint infection	i.v. and direct applplication	1 phage	5 × 10^9^	q24h	3 days	Yes	Resolution	Yes	Transient elevation in liver enzymes	2 mo
Exarchos et al., 2020 (46); 31740948	S. aureus, Cutibacterium acnes	41-yr-old man	Cardiovascular implantable electronic device and aortic graft infection	Direct instillation	2 phages	1 × 10^6^	q8h	14 days	Yes	Clinical cur e	Yes	None reported	12 mo
Aslam et al., 2019 (47); 30661974	S. aureus	65-yr-old male	Ventricular assist device infection	i.v.	3 phages	3 × 10^9^ i.v.	q12h	28 days	Yes	Clinical improvement within 1 wk; continued to be well appearing 7 mo later	Y es	None reported	7 mo
Mulzer et al., 2020 (48); 31651936	S. aureus	67-yr-old male	Ventricular assist device infection	Direct application	2 phages	2 × 10^7^	q8h	10 days	Yes	Clinical cure	Yes	Mild nausea	9 mo
Tkhilaishvili et al., 2021 (49); 34058260	P. aeruginosa	53-yr-old male	Ventricular assist device infection	i.v. and direct applplication	3 phages	5 × 10^9^	i.v. 7-h infusion direct q12h	5 days	Yes	Clinical cure; patient expired 4 mo later due to noninfectious cause	Yes	Mild nausea	4 mo
Rubalskii et al., 2020 (50); 32380707	S. aureus	45-yr-old male	Infusion pump infection	Direct application	1 phage	4 × 10^10^	Once	Once	Yes	Clinical cure	Yes	None reported	>2 yr
Rubalskii et al., 2020 (50); 32380707	Escherichia coli	66-yr-old female	Sternal infection after cardiac surgery	Direct application	2 phages	4 × 10^10^	Once	Once	Yes	Eradication of E. coli	Yes	None reported	2 yr
Rubalskii et al., 2020 (50); 32380707	P. aeruginosa	13-yr-old male	Sternal infection after lung transplant	Direct application	2 phages	4 × 10^10^	Once	Once	Yes	Resolution	Yes	None reported	2 yr
Rubalskii et al., 2020 (50); 32380707	S. aureus	51-yr-old male	Ventricular assist device infection	Direct application and oral	4 phages	1 × 10^9^	q12–24h	12 days	Yes	Microbiologic reduction of S. aureus	No	None reported	6 wk
Rubalskii et al., 2020 (50); 32380707	S. aureus	59-yr-old male	Aortic graft infection	Direct application	1 phage	1 × 10^9^	q12h	2 days	Yes	Clinical cure	Yes	None reported	>2 yr
Rubalskii et al., 2020 (50); 32380707	S. aureus	62-yr-old male	Ventricular assist device infection	Direct application	1 phage	1 × 10^9^	q12h	7 days	Yes	Clinical cure; died 20 mo after heart transplant due to transplant failure	Yes	None reported	20 mo
Rubalskii et al., 2020 (50); 32380707	K. pneumoniae	40-yr-old male	Pneumonia in settning of heart transplant	Nebulized and oral	2 phages	1 × 10^8^	q12–q24h	4 days	Yes	Clinicalcure	Yes	None reported	>3 yr
Rubalskii et al., 2020 (50); 32380707	S. aureus, Enterococcus faecium, P. aeruginosa	52-yr-old male	Aortic graft infection	Direct application and oral	4 phages	1 × 10^8^	One dose orally, direct instillation, and intraoperatively	1 day	Yes	Staphylococcus aureus, Enterococcus faecium, Pseudomonas aeruginosa not detected but died at 2 mo from a new bacterial infection	Yes	None reported	4 days
Chan et al., 2018 (51); 29588855	P. aeruginosa	76-yr-old male	Aortic graft infection	Direct application	1 phage	1 × 10^7^	Once	Once	Yes	Recovered	Yes	None reported	18 mo
Gilbey et al., 2019 (52); 31281964	S. aureus	65-yr-old male	Periprosthetic valve endocarditis	i.v.	3 phages	3 × 10^9^	q12h	14 days	Yes	Recovered and discharged home but on day 98 had progressive heart failure and a new potential vegetation with sterile blood cultures; declined surgery and died on day 103	Yes	None reported	103 days
Schooley et al., 2017 (54); 28807909	A. baumannii	68-yr-old male	Necrotizing pancreatitis with infected pancreatic pseudocysts	i.v. and direct application	9 phages (not all administered at the same time)	5 × 10^9^	q6–8h	∼12 wk	Yes	Recovered	Yes	None reported	>2 yr
Duplessis et al., 2018 (55); 28992111	P. aeruginosa	2-yr-old male	Endovascular infection and bacteremia	i.v.	2 phages	3.5 × 10^5^	q6	6 days	Yes	Sterilization of blood cultures; patient expired due to cardiac and septic shock attributed to undrained fluid collections	No	Decompensation attributed to heart failure but endotoxin release could not be excluded	None
Jennes et al., 2017 (57); 28583189	P. aeruginosa	61-yr-old male	Infected pressure sores	i.v. and direct delivery (50 mL phage solution irrigation)	2 phages	2 × 10^6^	i.v. q6h; direct delivery q8h	10 days	Yes	Pressure sores remained infected with intermittent episodes of sepsis	No	None reported	Died at 4 mo from Klebsiella pneumoniae sepsis
Fadlallah et al., 2015 (58); 26862360	S. aureus	65-yr-old female	Bacterial keratitis	Direct application	Not described	Not described	q12h	4 wk	Yes	Clinical resolution and negative ocular cultures	Yes	None reported	6 mo
Johri et al., 2021 (62); 34177601	P.	33-yr-old male	Prostatitis	Oral liquid, rectal suppositories, and urethral instillations	≥3 phages cocktails	Not described	Daily oral; BID suppositories; daily urethral	14 days oral; 10 days rectal; 10 d ays urethral followed by 2 mo of oral and rectal	No	Clinical and microbiological resolution	Yes	None reported	5 mo
Fish et al., 2018 (63); 30279396	S. aureus	63-yr-old female	Osteomyelitis	Direct application	1 phage	1 × 10^9^	Weekly	7 wk	Yes	Partial improvement	Yes	None reported	3 yr
Marza et al., 2006 (64); 16781080	P. aeruginosa	27-yr-old male	Skin graft infection	Direct application	1 phage	1 × 10^3^	Once	Once	Yes	Clinical improvement and negative cultures	Yes	None reported	3 days

a q12h, every 12 h; NA, not applicable; IL-6, interleukin-6.

### Recurrent UTIs.

There are anecdotal, uncontrolled reports of successful use of phage therapy (in combination with antibiotics) for treatment of patients with recurrent UTIs. Gut, vaginal, and urinary microbiomes are important reservoirs of uropathogens and likely contribute to the pathophysiology of recurrent UTIs (3, 4). In a patient with recurrent UTI secondary to MDR-K. pneumoniae, oral and intrarectally administered phage resulted in microbiologic clearance in urine and stool (5). In addition to potentially decreasing the gut reservoir of uropathogens, phages may decrease uropathogen colonization of urinary catheters. *In vitro* data suggest that phage-treated catheters can be protected from colonization with common uropathogens (6). Other case reports showing successful treatment of UTI using phages include a liver transplant recipient with recurrent extended-spectrum beta-lactamase (ESBL)-producing E. coli UTIs (7), a kidney transplant recipient with an ESBL-producing K. pneumoniae UTI (8), an extensively drug-resistant (XDR) K. pneumoniae UTI (9), and a refractory P. aeruginosa UTI in the setting of ureteral stents (10). A single randomized controlled trial investigating phage therapy in men presenting for transurethral resection of the prostate with complicated or recurrent UTI yielded unfavorable results (11). In this trial, 28 patients received intravesicular phage, 32 received placebo mechanical irrigations, and 37 received systemic antibiotics, with no differences in urine culture sterilization across the three groups. Phage therapy was not inferior to standard-of-care antibiotic treatment, and it also was not superior to placebo bladder irrigation. Absolute success rates were lowest in the phage (18% success) compared to the placebo (28% success) and antibiotic (35% success) groups. Low concentrations of phages (10^4^ to 10^5^ PFU per dose) and insufficient coverage of bacterial pathogens due to use of a fixed cocktail and/or poor phage survival in urine (influenced by pH and temperature) may have impacted this study.

### Chronic rhinosinusitis and otitis media.

Chronic and recurrent bacterial rhinosinusitis can be difficult to cure with conventional antibiotic approaches secondary to biofilm formation and pathogens with difficult-to-treat resistance patterns (12). In a pilot uncontrolled clinical trial of nine patients with chronic rhinosinusitis, intranasal phages were administered for up to 14 days. Treatment was well tolerated, and infection was eradicated in two patients (12). A phase I/II double-blinded, placebo-controlled trial was conducted in 2009 randomizing 24 patients with drug-resistant chronic refractory P. aeruginosa otitis media into phage or placebo arms; three patients in the phage arm had undetectable P. aeruginosa compared with none in the placebo group (13). A follow-up phase III study has not been conducted.

### Skin and soft-tissue infections.

Like rhinosinusitis, skin infections offer the possibility for topical phage administration (14, 15). However, in a clinical trial including 13 patients randomized to phage therapy alone and 14 randomized to standard of care alone, phage decreased the bacterial bioburden but at a slower rate than standard of care in P. aeruginosa*-*infected burn wounds. Low phage concentrations, lack of baseline PST, and poor adherence of phage to the dressings used may have contributed to the negative trial results (16). Radiation burn wounds infected with S. aureus (a highly specific infection type) were successfully treated with topical phage (Table 1) (17).

### Respiratory infections.

Recurrent infections with MDR Gram-negative bacilli are common in patients with CF as well as lung transplant recipients. There are case reports of phage therapy in CF patients with respiratory infections caused by MDR Gram-negative bacteria (18–22) (Table 1). In one such case, a patient with CF and worsening acute-on-chronic respiratory failure due to MDR P. aeruginosa despite antibiotic therapy improved with concomitant addition of four intravenous (i.v.) phages for 8 weeks, enabling the patient to undergo successful bilateral lung transplantation (20). Hoyle et al. published the experience of a teenager with CF and an MDR *Achromobacter* spp. pulmonary infection who received both nebulized phages once daily as well as oral phage twice daily periodically over a year, with improved lung function (22). Several other cases of apparent successful use of prolonged courses of i.v. and inhaled phage to treat respiratory infections in lung transplant patients have been reported (23–25) (Table 1). For example, a 15-year-old female with CF who had undergone bilateral lung transplantation and developed disseminated M. abscessus infection, including skin and soft-tissue and respiratory infection, was successfully treated using a three-phage cocktail, including an engineered phage, in addition to traditional anti-infective therapy (23). However, clinical failure was observed in an immunocompetent 81-year-old patient with refractory M. abscessus pulmonary disease in the setting of bronchiectasis, associated with development of a strong neutralizing antibody response (see Question 9) (25). A series of four patients with coronavirus disease 2019 (COVID-19) and secondary infection with A. baumannii were treated with nebulized phages, with clinical cure in two cases (26) (Table 1). Another non-CF case involved a patient with refractory P. aeruginosa pneumonia and empyema treated with i.v. and nebulized phages for 7 days, with clinical cure (27).

### Bone and joint infections.

Bacteria in biofilms possess a variety of mechanisms for immune and antibiotic evasion, making biofilm-related infections both difficult to detect and eradicate. Phages are a potential approach for treating biofilm-mediated musculoskeletal infections, especially when hardware cannot be removed. *In vitro* data indicate that some phages are active against biofilms of different ages (28) and with different extracellular matrix types (29).

Prior to the 21st century, there were several case series, primarily from Poland and the Republic of Georgia, describing use of phages for treatment of musculoskeletal infections, generally in conjunction with antibiotics, with clinical success described in upwards of 90% of cases (30, 31). Several clinical phage treatment experiences with musculoskeletal infections from diverse parts of the world have been published in recent years (Table 1). Specific musculoskeletal infections for which phage therapy has been used include PJI, spinal hardware infection, trauma-related injury associated with infected hardware, and craniectomy-related infection. Phage therapy has been administered both i.v. (7, 32–35) and locally to treat musculoskeletal infections (36–40). To highlight one case series, Onsea et al. describe their experience using phage therapy to treat four orthopedic infections, using a standardized treatment approach to locally administer phage (36). Phages were administered intra- and postoperatively three times daily for a maximum of 10 days. All patients also received antibiotics; their clinical status was monitored daily during phage therapy. There were no recurrences of infection over 8 to 16 months of follow-up (36).

### PJI.

Several case reports employed local instillations of phages in patients with chronic S. aureus, Staphylococcus epidermidis, or P. aeruginosa PJI, with phages administered in conjunction with debridement, antibiotics, and implant retention (DAIR). This approach has yielded successful outcomes (Table 1) in patients who were not candidates for resection arthroplasty, allowing implant salvage while controlling or curing the infection (37–39, 41–43). Another case reported clinical cure of chronic K. pneumoniae PJI with an i.v. phage without surgical debridement (34). Other reports describe successful treatment of chronic S. aureus PJI with resection arthroplasty (44, 45).

### Cardiac device-associated infection.

Cardiac infections for which phage therapy have been administered include infections associated with cardiovascular implantable electronic devices (CIEDs) (46), ventricular assist devices (VADs) (7, 47–50), vascular grafts (50, 51), and prosthetic valves (52). Several reports of CIED infections treated with phages have been described, generally due to S. aureus or P. aeruginosa (Table 1). A series of eight cases reported by Rubalskii et al. included infected vascular grafts, infected VADs, and sternotomy infections postcardiac surgery, caused by S. aureus, K. pneumoniae, E. coli, and P. aeruginosa; clinical resolution was achieved in seven of eight cases (50). In other reports, two cases of S. aureus and one case of P. aeruginosa VAD infection were successfully resolved with phage therapy in conjunction with antibiotics (47–49); one patient with S. aureus infection was treated with i.v. phages for 4 weeks in addition to systemic antibiotics and the other with local instillation of phage through an indwelling drain for 10 days, alongside systemic antibiotics, following debridement. The patient infected with P. aeruginosa was given a 7-h i.v. infusion of phages, followed by local application during surgical debridement, and then local instillation through an indwelling drain every 12 h for 5 days. Another patient with extensive local CIED infection caused by S. aureus associated with a bypass graft infection was successfully treated with debridement followed by local phage instillation for 2 weeks (46). However, two patients with P. aeruginosa VAD infections treated with i.v. phages for six to eight weeks, along with i.v. antibiotics, experienced infection recurrence (7). Gilbey et al. reported treating a patient with S. aureus aortic valve endocarditis with i.v. phages for 14 days in addition to antibiotics; he recovered and was discharged but returned 3 months later with progressive heart failure, potential vegetations, and negative blood cultures (52).

### Sepsis.

As with all infections, the role of phage therapy in acute sepsis is unclear. In 2003, Weber-Dabrowska et al. reported observations in 94 septic patients failing antibiotic therapy with a mixture of monomicrobial and polymicrobial infections from a variety of syndromes, including UTI, skin and soft-tissue infection, respiratory infection, and intra-abdominal infection (53). Phage therapy was generally administered orally three times per day for a median of 29 days and was given with antibiotics in 71 subjects. Recovery was achieved in approximately 85% of cases; there was no statistically significant difference between the antibiotics plus phage and phage monotherapy groups (53). In a separate study, a 68-year-old diabetic patient received phage therapy to treat necrotizing pancreatitis complicated by an MDR A. baumannii-infected pancreatic pseudocyst. The patient, who was comatose and septic, was successfully treated with phage therapy administered both i.v. and via percutaneous catheters directly into the infected cavities, showing significant improvement within 48 h of phage administration (54). A 2-year-old patient with DiGeorge syndrome (Table 1) who had a mycotic aneurysm due to P. aeruginosa was given i.v. phage therapy with sterilization of blood cultures but ultimately succumbed to sepsis, attributed to undrained fluid collections and lack of surgical source control (55).

### Gaps in knowledge.

There is a near-total lack of randomized controlled trials of phage therapy; such trials, which should use standardized dosing regimens, are urgently needed. Further analysis of factors associated with success and failure will be necessary to develop an understanding of the role of phages as treatments for bacterial infections. Analysis and publication of studies with negative outcomes is essential for advancement of this understanding. Many questions remain about selection of cases, treatment indications, stages of illness, and acuity/chronicity of illness. In addition to clinical trials, a systematic approach to data collection from compassionate use cases and availability of such data to clinicians, including clinical failures, would be helpful.

## QUESTION 2: SHOULD ANTIBIOTICS BE ADMINISTERED CONCURRENTLY WITH PHAGES?

### Suggestion.

The ARLG Phage Taskforce suggests that if phage therapy is used, it should be in conjunction with conventional antibiotics.

### Rationale.

Recent clinical data on phage therapy has been generated primarily in compassionate use settings in conjunction with antibiotic therapy (7, 9, 20–27, 32–35, 37–39, 41–47, 50, 51, 54, 56–58). Many cases were associated with apparent successful response of MDR and/or biofilm-associated infections that were not resolving with antibiotics alone, suggesting that in those cases, there may have been an additive or synergistic effect of the phage-antibiotic combination (9). An additional benefit of using phages in combination with antibiotics is the potential to reinstate susceptibility of the targeted bacteria to antibiotics by manipulating bacterium-phage coevolutionary strategies. In one study, the investigators collected bacterial isolates before and after phage treatment and observed changes in antibiotic susceptibility patterns and a reduction in pathogen fitness after treatment with phages (54). Chan et al. used a phage known to effect an evolutionary trade-off between phage resistance and antibiotic susceptibility *in vitro* (59) to successfully treat a patient with a P. aeruginosa-infected aortic graft (51). These clinical findings are supported by *in vitro* and animal model data demonstrating phage-antibiotic synergy in some circumstances (see Question 8).

### Gaps in knowledge.

There is a paucity of controlled clinical trial data on the effectiveness of phage-antibiotic combinations. The panel suggests that bacterial isolates before and after phage therapy be tested for antibiotic and phage susceptibility, despite limitations of the latter (see Question 7). Further research is needed to investigate the potential for attenuated bacterial virulence after initiation of phage therapy. Clinical trials comparing antibiotic alone versus antibiotic plus phages are needed to determine the value of phage therapy over antibiotic therapy alone; assuming phage plus antibiotic therapy is demonstrated to be active in such trials, subsequent trials might consider assessing phage plus antibiotics versus phage alone.

## QUESTION 3: IS PHAGE THERAPY SAFE FOR CLINICAL USE AS AN ANTI-INFECTIVE?

### Suggestion.

The ARLG Phage Taskforce determined that phage therapy is generally safe to administer, with adverse events rarely reported. Taskforce members suggest that patients receiving their first dose of phages be observed for allergic and other adverse reactions and that monitoring of renal and liver function, as well as a complete blood count (CBC), take place on a weekly basis at minimum until robust safety data are established.

### Rationale.

Phage therapy is considered generally safe, with relatively few reported adverse events (AEs), provided that the phage preparation administered meets Good Manufacturing Practice (GMP) or similar regulatory criteria (60, 61). Most published accounts of phage therapy report no AEs after phage administration via oral (5, 8, 22, 50, 62), local (9, 36–42, 46, 48, 50, 51, 54, 57, 63, 64), inhaled (22, 24, 26, 27, 50), or i.v. (7, 20, 21, 25, 27, 32, 34, 44, 47, 52, 56, 65) administration. During clinical use of phages, transient AEs have been observed, but their association with the administered phages has oftentimes been unclear. AEs reported in the literature include the following. An 82-year-old male developed fever, shortness of breath, and wheezing after two infusions of high doses (1 × 10^11^ PFU/ml) of phages; symptoms resolved with acetaminophen, methylprednisolone, nebulized albuterol, and diphenhydramine, and he continued phage therapy at a lower dose without further incident (7). In a separate report, 2 h after a first phage therapy dose, a patient with an infected craniotomy site became briefly hypotensive; no treatment was required, and the hypotension resolved spontaneously (33). A patient with disseminated M. abscessus treated with engineered phage had sweats and flushing for the first 2 days of therapy but continued therapy without event (23). A 72-year-old male patient with S. aureus PJI developed reversible transaminitis after three doses of an i.v. phage (45). A 42-year-old male with P. aeruginosa bacteremia experienced fever and chills on the third day of therapy for a UTI, which resolved 48 h after phage therapy was discontinued (66). Finally, a 7-year-old girl developed fever and transient increase of pain at the infected site (her heel) after receiving a first dose of i.v. phage (35).

Phage therapy clinical trials provide further AE data. In a single-arm safety trial of i.v. staphylococcal phage cocktail conducted in Australia, 13 patients with S. aureus bacteremia received phage therapy with no AEs reported (56). In a clinical trial assessing outcomes of topical phage therapy versus sulfadiazine silver on infected burn wounds, one death occurred in each study arm and mild AEs were observed but were deemed unrelated to phage treatment (16). In a randomized, double-blinded, placebo-controlled trial of chronic otitis media (Question 1), no serious adverse events were reported and all treatment-emergent adverse events were mild to moderate and considered not related to phage administration (13). Finally, in an open-label, uncontrolled trial of phage nasal irrigation, six mild, treatment-emergent, self-resolving AEs were observed, including diarrhea, epistaxis, oropharyngeal pain, cough, rhinalgia, and decreased blood bicarbonate. Taken together, the evidence suggests that phage therapy is generally safe, but because of incomplete data as to the safety profile of phage therapy, the ARLG Phage Taskforce suggests that patients be monitored, at least during the first dose of phage administration, for allergic or other reactions, as further described in Question 4.

## QUESTION 4: WHAT ARE SOME PRACTICAL CONSIDERATIONS WHEN CONSIDERING PHAGES AS ANTI-INFECTIVE THERAPY?

### Suggestion.

The ARLG Phage Taskforce suggests that a detailed plan outlining uncertain clinical outcomes, lack of proven efficacy, lack of standardized dosing or administration, potential adverse events, costs, and other logistics be discussed with patients as part of the informed consent process before administering phage therapy.

### Rationale.

Several commercial and academic entities can potentially source, characterize, and biomanufacture phages for compassionate use. When engaging with these entities, two key considerations determine whether a patient is a phage candidate. First, the bacterial isolate is needed to identify phage(s) with lytic activity against the isolate. Asking the clinical microbiology laboratory to store the patient’s bacterial isolate(s) allows the possibility of subsequent screening for identification of appropriate phages.

Second, the clinical status of the patient is important in determining eligibility for expanded access to phage therapy. Critically ill patients in an intensive care setting generally need therapy within hours or days, and this is not frequently possible with phage therapy, given the delay between candidate identification to delivery of a clinical-grade phage preparation. The median time from request to phage administration ranges from 28 to 386 days, with a median of 171 days (7). However, the process can possibly be expedited for administration of phages to critically ill patients, as shown in a study of secondary bacterial pneumonia as a sequela of COVID-19 infection (26).

Since phage therapy is not U.S. Food and Drug Administration (FDA) approved, it is not reimbursable by insurance. In the expanded-access pathway, phage preparations are frequently provided *pro bono*. However, due to rapidly rising requests for phage therapy, several academic laboratories are now charging for phage preparation. Costs may be associated with administration at outpatient infusion centers, including administration fees, travel, and board. Costs such as those of a peripherally inserted central catheter (PICC) or a nebulizer, if indicated, as well as of concomitant antibiotic administration and routine blood tests, are generally billed to the patient’s medical insurance, as they are considered standard of care.

As discussed in Question 3, the first dose of phage therapy should be administered in the presence of a health care provider so that a careful assessment for immediate AEs, such as flushing, rash, and breathing difficulties, can be recognized and appropriately treated. Safe administration by the patient (or a trained companion) has been documented for i.v. (7) and nebulized (67) phages. In the home setting, extensive patient education is needed, with clear instructions on phage storage and use. Additionally, there should be a plan for prospectively managing AEs and ongoing surveillance of renal and liver function, as well as a CBC, on at least a weekly schedule. For i.v. phage administration, central line or PICC placement is not a requirement but is often convenient for long treatment courses.

A detailed discussion as to the experimental nature of phage therapy and the lack of efficacy data in clinical trials should take place with the patient before initiation of phage therapy. The conversation should address what the patient hopes to achieve from phage therapy and can frame the discussion regarding goals of care. Phage therapy has not been proven to provide a therapeutic benefit; thus, it should be reinforced that there is a likelihood that patients may experience no improvement in their clinical outcome with phage therapy. A dialog about the safety record of phages should be factored into a risk-benefit discussion. The optimal clinical indications, duration of therapy, dose, frequency of dosing, and concurrent use of antibiotics have yet to be defined. Clinical trials are ongoing to lend clarity to these unresolved questions, but until they are completed, patients should be aware of the highly experimental nature and unproven success of phage therapy.

## QUESTION 5: WHICH REGULATIONS GOVERN USE OF PHAGES IN CLINICAL SETTINGS?

### Suggestion.

The ARLG Phage Taskforce suggests that expanded access (commonly known as compassionate use) is a viable regulatory pathway for treatment of individual patients with phage therapy. The ARLG Phage Taskforce also endorses the concept of a common database to collect systematic data on patients who receive phage therapy under the expanded-access pathway until and if FDA approval of a licensed phage therapy is achieved.

### Rationale.

In the United States, phages intended for clinical therapeutic use are regulated as biological products by the Center for Biologics Evaluation and Research (CBER) at the FDA (68, 69). Currently, the FDA has not approved any phage product for clinical use, including treatment of bacterial infections. Therefore, with limited exceptions, United States investigators or clinicians who intend to administer phage to patients must first submit an investigational new drug (IND) application to the FDA.

INDs may be submitted by industry or research sponsors as part of prelicensure clinical trials. IND submissions must include extensive data on animal studies and toxicity; manufacturing, chemistry, and controls; data from any prior human research; investigator information; and study protocols for the intended clinical trial(s) (70). The IND must contain sufficient information to ensure “proper identification, quality, purity, and strength” of the investigational drug (71). At the time of this writing, multiple phase I and II clinical phage trials are or will soon be recruiting patients at U.S. study sites (https://www.clinicaltrials.gov/ct2/results?term=bacteriophage+AND+bacteriophage+therapy&cntry=US).

In the United States, the primary route for a patient to access an investigational product such as phage therapy is to enroll in a clinical trial. For patients who cannot access or do not qualify for clinical trials, there are other pathways for accessing investigational products. The most common pathway is expanded access, which encompasses the use of an investigational product such as phages, where the primary purpose is to monitor, diagnose, or treat patient(s) rather than to obtain effectiveness and safety data to support licensure. The expanded-access pathway is often referred to in the clinical literature as compassionate use. Expanded access requires that (i) a patient have a serious or immediately life-threatening condition or disease; (ii) no comparable or satisfactory alternative therapy options are available; (iii) the potential benefit justifies the potential risks of use; and (iv) providing the product through the expanded access pathway would not interfere with or otherwise compromise necessary clinical investigations of the product to support licensure (73).

Multiple categories of expanded access exist and are based on the size of the intended treatment population. The first category, individual patient expanded access, is the mechanism used in most published case studies of phage therapy. Physicians applying to treat patients under the expanded-access pathway submit FDA Form 3926, available online (74). Necessary data include a summary of the patient’s clinical history and rationale for expanded-access treatment; intended treatment plan; product manufacturing information; and safety and adverse event monitoring plans (73). For phages specifically, the FDA recommends the following information be provided: phage source, titer, endotoxin content, sterility, and test results of the preparation’s activity against the patient’s bacterial strain or strains (60). Assuming the FDA does not place a clinical hold on the application, treatment can begin 30 days after the application is received by the FDA or upon earlier notification (73). In emergency situations, the FDA may authorize expanded-access use (including by telephone) without a prior written submission. While institutional review board (IRB) approval is not required before emergency treatment, the IRB must be notified within five working days of treatment initiation. A full written submission must also be submitted to the FDA within 15 working days of emergency authorization (75). Following expanded-access treatment, a written summary of the results of expanded-access use, including AEs, must be submitted to the FDA, along with a brief annual report (73, 75–77). Additionally, any unexpected fatal or life-threatening suspected adverse reactions of treatment must be reported within 7 days (73, 75–77). Historically, the FDA has allowed >99% of expanded-access treatment applications for individual patients to proceed (78). Although expanded access plays a role in providing seriously ill patients with otherwise unavailable therapies, it is not a substitute for rigorous clinical testing and regulatory approval. To ultimately approve products for phage therapy, the FDA will need to determine that they are “safe, pure, and potent” (69), in other words, that they are safe and effective for their proposed use (79).

## QUESTION 6: UNDER WHICH CONDITIONS SHOULD PST BE USED TO SELECT PHAGES FOR THERAPEUTIC USE?

### Suggestion.

While it would be ideal to perform PST before phage administration so that a phage or phages active against the infecting bacterium is or are selected for use, standardized, accurate, and reproducible methods, reported with validated interpretive criteria are lacking (see Question 7). Once such methods are available, their routine use is suggested before phage administration, when possible.

### Rationale.

Phages do not have reliable activity against all strains of any bacterial species, underscoring the potential importance of PST to identify phages active against the infecting pathogen before administration. Once standardized PST methodologies become available, members of the ARLG Phage Taskforce suggest that attempts be made to perform PST before phage administration where possible. Furthermore, because of the potential for resistance to develop to phage during treatment (26, 54), confirmation of the continued activity of phages against the bacterial pathogen is likely to be helpful to determine whether clinical failures may be due to emergence of phage resistance or alternative reasons, such as a need for source control. Evidence suggests that infections caused by pathogens that develop resistance to a phage cocktail are not effectively treated unless a new phage or phage cocktail that shows activity against the resistant isolate is administered (26, 54).

PST may involve testing of a panel of phages (sometimes referred to as generating a phagogram [7]) to select one or more phage for therapeutic use. Current approaches to determining phage susceptibility are described in Question 7. There is potential dynamic tension with the time testing may take to perform, especially as there is no available method that can be easily incorporated into clinical microbiology laboratory workflows and because testing may need to occur offsite. Phage cocktails or phages with broad host ranges may allow empirical phage therapy in some emergency situations; however, confirming phage activity is still considered ideal before initiation of therapy, once suitable PST methods become available.

### Gaps in knowledge.

There is a lack of a standardized clinically available method for PST and correlation between *in vitro* activity and clinical efficacy. This is unfortunately at odds with making a recommendation to perform PST, since interpretation of results of such testing is not standardized. When phage cocktails are administered, approaches to testing for potential synergy or antagonism between phages may be helpful, although with conventional antibiotics, this type of testing is rarely performed clinically. Further, there is a need to understand phage activity against bacteria in biofilms (80); whether or not biofilm PST might be clinically useful is unknown (as there are no standardized methods), but notably this type of testing is not performed with conventional antibiotics.

## QUESTION 7: WHICH PARAMETERS SHOULD EMERGING PST PLATFORMS CONSIDER?

### Suggestion.

The ARLG Phage Taskforce identified several laboratory testing strategies for assessing phage activity against individual bacterial isolates, with no reference gold standard method identified. Methods ideally need to be standardized, accurate, reproducible, rapid, test multiple phages at a time, and report out using interpretive criteria that predict clinical activity; no such criteria yet exist. The ARLG Taskforce suggests that methods that demonstrate lack of *in vitro* phage activity against the targeted bacterium be shown to correlate with unlikely *in vivo* activity.

### Rationale.

Members of the ARLG Phage Taskforce reviewed common laboratory methods used for PST (sometimes referred to as host range testing), which have been applied to identify lytic phages potentially suitable for phage therapy. Two common approaches are double-layer agar and liquid testing methods.

With the double-layer agar method, a bacterium-phage mixture in melted low-concentration agar is spread over a solid agar nutrient medium in a petri dish (81). The semisolid state of the bacterium-phage layer restricts movement of bacteria and phages. The plate is incubated at a defined temperature for a defined period, dependent on the bacterial species tested (e.g., 18 to 24 h), although such criteria have not been standardized. Bacteria multiply, producing a confluent lawn of bacteria in the top agar layer. Lytic phages can infect the bacteria, replicate, cause cell lysis, and produce progeny, which infect neighboring bacterial cells. The cycle of infection and lysis continues, killing bacteria in localized areas and ultimately creating plaques, with each plaque typically being the result of infective proliferation of one lytic phage (82). While larger clear plaques may represent greater effects than smaller plaques, plaque size may vary by phage and/or host. Turbid plaques may result from slight growth or formation of lysogens (Question 10). Limitations to this method include poor reproducibility, protracted turnaround time (83, 84), and inability to assess individual members of a phage cocktail (if tested together). Further, microbiological detection of phage activity only reflects the number of phages able to infect the host under select time constraints and to generate plaques visible to the naked eye, potentially missing phage subpopulations with clinically relevant biological or immunologic properties, such as phage immunoreactivity (85). Ion and metal content, agar concentration, the presence of organic compounds or detergents, specific antibodies, complement system elements, other phages, bacterial host age and growth phase, incubation temperature, and storage vessel may affect a phage’s infection capability (84–87). The spot test is a modification of the double-layer agar method that involves spotting a phage suspension onto a solidified agar base over which bacteria in melted low-concentration agar has been placed (88). After overnight incubation, susceptibility to phage is determined by observing plaques at sites of spotting. A limitation of this approach is that plaques on the spot test can result from “lysis from without” due to phage lysins in phage lysate material and therefore may not necessarily reflect phage propagation (or “lysis from within”) (89). Although these methods require minimal instrumentation and equipment, they can be time and material intensive.

Another approach to PST involves liquid testing, with phage added to a bacterial inoculum, ideally at a defined ratio (known as the multiplicity of infection [MOI]). Liquid-based testing captures longitudinal, semiquantitative data addressing phage-mediated changes to bacteria (e.g., bacterial metabolism or bacterial abundance assessed by optical density). Susceptibility is determined by comparison to behavior of the bacterium without phage. This approach may be amenable to standardized, automated, scalable testing of phage activity and theoretically may be used to simultaneously test antibiotics and even to assess synergism or antagonism of combination therapies. Liquid-based assays furnish time-based measurements; theoretically, the longer the phage affects a bacterium, the greater the phage effect and/or the lower the chance for emergence of phage resistance may be. If this is the case, the ideal length of time needed to carry out such assays needs to be defined. That said, these theoretical possibilities have yet to be shown to be clinically relevant.

### Gaps in knowledge.

There is no reference standard method for PST. *In vitro* parameters that will predict undefined clinical efficacy are unknown. There is a need for standardized, reproducible, rapid, high-throughput methods for PST. Assays that measure plaque formation or growth profiles of bacteria in the presence of phages have been used; whether differences in plaque morphology or growth profiles can assess degrees of activity in a clinically meaningful way is undefined, as are thresholds for activity versus no activity. Whether liquid-based tests will have a greater ability than agar methods to identify phages or phage cocktails capable of mitigating phage resistance remains to be determined.

Whether results of PST should be reported as “active” and “inactive” or possibly, like antibiotics, “susceptible,” “intermediate,” and “resistant” is undefined. Further, it is unclear whether universal breakpoints can be applied for all phage-bacterium combinations or whether criteria might vary by bacterial species and/or phage type. Whether different testing or interpretive criteria should be applied for initial versus on-therapy testing is also unknown. Whether there exist benefits to testing cocktails versus monophage is unknown, as is how to test phage cocktails and whether custom versus fixed cocktails should be tested; for phage cocktail testing, each combination may be unique (to the individual patient’s bacterium) so that generalization may prove challenging.

Standardization of phage concentrations, media and agar compositions and concentrations (if relevant), incubation temperatures and durations thereof, bacterial densities and growth phases, quality control, and results interpretation are needed so that reproducible, accurate methods can be applied in clinical microbiology laboratories. Ideal phage relative to bacterial host concentrations deserve consideration in emerging methods assessing phage activity. Methods to quantify phages used for laboratory testing ideally should be able to distinguish viable from nonviable phages. Idealized quality control processes for testing phage activity have yet to be established; this is particularly challenging given the biological nature (and potential for evolution) of phages. Phages assessed must be standardized (e.g., from a phage bank stock of known titer) and processes put into place to mitigate changes in that stock (e.g., viability, concentration, mutation) over time. This may be particularly challenging in the case of newly isolated phages. Phages, like certain antibiotics, adsorb to certain surface types, such that labware used to handle them may affect their amounts, an effect potentially mitigated by the addition of surfactant (Tween 20) or plasma (90).

With all approaches, storage conditions may adversely affect phage enumeration (91–93), and specificity needs to be considered, as phages can be present naturally. Ideally, novel assays of phage activity will identify improved phage storage conditions to optimize their stability and exhibit low between- and within-run variability independent of reagent brands and temperatures. Moreover, improved assays should account for the impact of relative phage concentration on observed bacterial reduction, such that *in vitro* results can be extrapolated to predict clinical effect.

Bacterial and phage sequencing as a proxy for phenotypic testing could be explored. Genetic testing potentially can open new avenues for selection of appropriate therapeutic phages or cocktails, including methods to test for the likelihood of emergence of resistance, such as those described in reference 94.

Finally, although it might be assumed that lack of *in vitro* phage activity against a particular bacterium will imply poor clinical outcome, phages may have enhanced activity in particular microenvironments in which they interact with their bacterial hosts, and such environments may not be adequately represented *in vitro* (95). This could involve phage adaptations that specifically contend with the microenvironment and/or a physiologic state of the bacteria that may be more conducive to being predated by lytic phages in particular microenvironments. Conversely, demonstration of *in vitro* activity may not be predictive of *in vivo* activity because of the complex biology involved.

## QUESTION 8: WHICH PARAMETERS SHOULD TEST METHODS FOR ASSESSMENT OF PHAGE ACTIVITY IN COMBINATION WITH ANTIBIOTICS CONSIDER?

### Suggestion.

As with PST, the ARLG Phage Taskforce found no standard method for phage-antibiotic combination testing. Such an assay may be helpful to predict effects of specific phage-antibiotic combinations on bacterial population reductions, addressing synergy and antagonism.

### Rationale.

As discussed in Question 2, most recent clinical data on phage therapy have been generated when used in conjunction with antibiotics, making it difficult to draw conclusions as to the relative contributions of phages and antibiotics to outcomes. *In vitro* studies indicate that some phages lower some antibiotics’ MICs (96, 97), with phage-antibiotic synergy demonstrated under certain circumstances (98–100). Thus, methods for ascertaining combination activity may inform selection of specific phage-antibiotic combinations.

A systematic assessment of activity of phages combined with antibiotics against E. coli revealed diverse responses, ranging from additive to synergistic to antagonistic effects, dependent on antibiotic class and resistance profile of the bacterial strain (9, 101). Some studies examining phage-antibiotic interactions in the presence of host factors such as urine and bile salts demonstrate synergy (102), whereas others do not (101, 103). Animal studies have demonstrated additive or even synergistic effects of certain phage-antibiotic combinations (104–107). Phage-adjuvanted antibiotic effects were observed in an E. faecalis sepsis model, suggesting that, under some circumstances, lower doses of antibiotics are acceptable when combined with phages (104). In the case of antagonism, phage sequencing may be useful to compare the similarity of ribosomal genes or DNA polymerase to those of the bacterial host; if they are similar, antibiotics that inhibit these may impact the phage life cycle.

There is evidence that phage-antibiotic combinations can reduce the likelihood of emergence of antibiotic- and phage resistant-bacteria and decrease bacterial fitness (54). In a study using over 400 A. baumannii isolates, there was a correlation between antibiotic resistance and phage susceptibility, suggesting evolutionary trade-offs (108). Phage-plus-antibiotic combinations reduced phage and/or antibiotic resistance of S. aureus, E. coli, P. aeruginosa, and *Enterococcus* species *in vitro* (101, 109–114). Phage specificity is related to phage-receptor interactions (115), with internal bacterial cellular functions (e.g., gene expression and DNA replication) also playing a role (115). Some phages use virulence factors (116) or antibiotic resistance proteins (59) as receptors, such that bacteria that develop resistance to the phages via receptor mutation may be less virulent or more antibiotic susceptible, respectively.

Phage-antibiotic synergy has also been observed in biofilms. When tested against monospecies or mixed biofilms, phages combined with antibiotics reduced bacterial densities of P. aeruginosa, S. aureus, and enterococcal biofilms *in vitro* (117–121), with enhanced activity if treatment with phages preceded antibiotics (98, 122). Similar results were observed against P. aeruginosa from CF airways and wounds (123). In an animal model of orthopedic implant-related infection, S. aureus but not P. aeruginosa biofilm thickness was reduced following phage-antibiotic combinations more so than either alone, suggesting that bacterial species, alongside infection type or site, impacts effects of combination activity (124).

Emerging methods might consider consolidating phage and antibiotic susceptibility testing methods in a way that could allow for combination testing, for example, using a checkerboard assay in which various antibiotics are added to wells of a liquid testing plate (see Question 7); this may allow exposure of targeted bacteria to variable concentrations of antimicrobials and single or multiple phages (16, 125, 126). Gu Liu et al. offer a method for phage-antibiotic testing using 96-well plates that could be developed for use in clinical microbiology laboratories (101) to assess phage-antibiotic antagonism or synergy. Results of such testing need to be correlated with successful versus unsuccessful outcomes in clinical trials. Modified disk diffusion methods and time-kill analyses have been used to assess potential phage-antibiotic synergy (127).

### Gaps in knowledge.

An absence of controlled clinical trials data that incorporate phage-antibiotic testing was identified. Whereas there is evidence for phage-antibiotic synergy or antagonism in some cases, molecular mechanisms underlying such interactions are generally not well understood. It is unknown whether entire classes of antibiotics will demonstrate the same phage interactions as their constituent members. Likewise, it is unclear which phage species might best be paired with distinct antibiotic classes. As phage science progresses, presently unavailable mechanistic information may facilitate more rational phage-antibiotic pairings. Ultimately, each phage-antibiotic combination effect may be so unique across clinical isolates that generalization may be challenging. Additionally, whether testing of phage-antibiotic combinations (in addition to phage and antibiotics individually) against biofilms should be performed for biofilm-associated infections is undefined (Question 6).

## QUESTION 9: WHICH IMMUNE SYSTEM COMPONENTS ARE LIKELY TO IMPACT SAFETY AND EFFICACY OF PHAGE THERAPY, AND HOW CAN THESE BE TESTED?

### Suggestion.

The ARLG Phage Taskforce is unable to recommend specific assessment of immunologic parameters that correlate with phage activity that should be assessed due to a significant knowledge gap in this area but suggests considering tests for neutralizing antibodies in the context of prolonged phage administration, with the recognition that standardized assays for such measurements are unavailable. As indicated in Question 3, monitoring of renal, liver, and hematologic function is recommended; it may also be reasonable to monitor an inflammatory marker (e.g., C-reactive protein).

### Rationale.

Although bacteria are the designated hosts of phages, a complex interplay between the human (superhost) immune system, phages, and bacteria likely impacts phage activity and potentially larger aspects of human health and disease (128, 129). Few reports in the English literature have explored the nature of the immune response to phage therapy, although there is some evidence of synergy between the innate immune system and phages during therapy, with phagocytosis possessing a major role. Early work showed phages to be cleared by the reticuloendothelial system, with deposition in the spleen (130). A recent study showed lower phage concentrations in spleens of mice with extant LPS-induced systemic inflammatory responses and more effective phage inactivation by splenocytes from these compared to control mice (87). Roach et al. reported that neutrophils and innate immune cell signaling impact effective phage treatment of acute pneumonia in mice (131). In a recent report on the treatment of a 7-year-old girl with P. aeruginosa septic arthritis and osteomyelitis (see Question 1), there was upregulation of genes associated with innate and adaptive immunity in response to i.v. phage therapy (35).

Phages have been observed to reduce markers of inflammation, such as C-reactive protein, with treatment (132). Results of a recent study of severe S. aureus infection, including infective endocarditis and septic shock in humans, suggest that phage therapy induces an anti-inflammatory response; inflammatory markers decreased in 11 of 13 patients, and transcriptome analysis indicated changes in regulation of genes involved in the innate immune response in the blood of infective endocarditis patients following phage administration (56). However, a separate study of cytokine expression following human monocyte-derived dendritic cell treatment with S. aureus phage K found that phages had little impact on pro- and anti-inflammatory cytokine or CD80/CD86 and major histocompatibility complex class I/II protein expression (133). Because phage preparations may contain remnants of bacteria used for phage propagation, it may be difficult to distinguish immune responses elicited by phages from those elicited by bacterial components in phage preparations. A reasonable approach is to consider monitoring C-reactive protein with prolonged phage therapy, especially systemic administration.

Antibody responses to phages have been reported to develop over the course of phage therapy. Factors that contribute to levels and classes of antibodies generated include the route, dose, and frequency of administration. Studies in mice and humans have found weak antibody responses to be induced after oral administration (134–136). Majewska et al. found that although phage-specific IgG antibodies were detected in blood early during prolonged oral phage treatment, only secreted IgA in feces, detected in late treatment, coincided with decreased phage levels (136). Arguably, antibodies may not be highly relevant to topical phage applications. In mice treated intraperitoneally (i.p.) with phages, antibody responses appeared to have an impact on phage levels, with a decrease in circulating phages in mice immunized with phages before subsequent treatment (87, 137). However, despite decreased phage levels, treatment was nonetheless effective in reducing bacterial burden and wound size in a murine model, albeit not to the extent of unimmunized treated mice (137). Similarly, the presence of phage-specific antibodies may not impede effective therapy in humans, as reported in 20 patients who received an antistaphylococcal phage cocktail (135). On the other hand, Dedrick et al. recently reported on a patient with bronchiectasis and M. abscessus pulmonary infection (see Question 1) who initially responded to an i.v. 3-phage cocktail with decreased M. abscessus counts in sputum but, with continued treatment, developed a neutralizing antibody response that corresponded to increased disease burden and treatment failure (25). The ARLG Phage Taskforce suggests considering measurement of neutralizing antibodies with prolonged courses of phage therapy but recognizes that no standardized assays exist to measure neutralizing antibodies.

### Gaps in knowledge.

Although immune responses, such as changes in inflammatory responses and antiphage antibodies, have been observed in some studies, controlled clinical studies are needed to determine whether these phenomena are consistently observed during human phage therapy and whether they vary depending on immune status of the patient and are clinically relevant. Further, the impact of immune response on therapeutic efficacy needs to be assessed in patients receiving phage therapy. Another factor to consider is that the diversity of phages used therapeutically may result in variable immune responses from one phage to another. Whether immunologic effects are relevant in various types of immunocompromised patients is unclear, although immunocompromised patients have undergone treatment with phages with no observed adverse events and apparent clinical success (138). While concern has been expressed about the potential effect of preexisting immunity to phages due to environmental phage exposure, there is insufficient evidence to determine whether this will have an impact on phage therapy.

If assessing for evidence of phage neutralization in patient specimens (serum for systemic administration and possibly respiratory secretions for inhaled administration) in the context of prolonged phage administration in clinical trials, testing might be conducted before treatment initiation (or early on, e.g., before 2 weeks of therapy) and after two or more weeks of therapy to assess whether phage treatment failure in later weeks might be due to neutralization of phage activity in the patient. If no evidence of neutralization is observed, phage therapy could continue with the same phage preparation. If phage neutralization is observed, a secondary phage preparation distinct from the original phage preparation (as determined by genomic comparison and neutralization testing) might be considered. Such findings could be correlated with phage levels and clinical outcomes. Notably, there are no standardized assays for measurement of neutralizing phage antibodies available, so if such testing is ultimately shown to be clinically helpful, standardized assays will be needed.

Whether or not allergic responses occur in association with phage therapy is unknown (but does not appear to be common); if allergic responses become a concern, measurement of biomarkers that predict such responses could be considered for study.

## QUESTION 10: WHAT ARE CURRENT ACCEPTABLE STANDARDS NEEDED FOR SAFE PHAGE ADMINISTRATION?

### Suggestion.

The ARLG Phage Taskforce suggests that phages used for phage therapy should not encode antibiotic resistance or toxin genes in their genomes and should not be capable of undergoing lysogeny. Phages should be sequenced to demonstrate absence of identifiable antibiotic resistance elements, bacterial toxin genes, integrase genes, regulators of integrase genes, and integrase-like genomic elements in their genome. Bacterial hosts used for phage propagation should also ideally be sequenced and shown not to harbor toxin and antibiotic resistance genes. Phage therapy formulations should be sterile according to U.S. Pharmacopeia (USP) 71 and tested in College of American Pathologists (CAP)- or Clinical Laboratory Improvement Amendments (CLIA)-certified laboratories to confirm the presence of low levels of endotoxin and secreted bacterial products.

### Rationale.

As with any medical intervention, the first consideration is safety. Primary safety concerns include phage predilection to lysogeny and formulation sterility and mitigation of secreted bacterial products.

The history of the phage and bacterial propagation host should be well-documented. Ideally, annotated whole-genome sequences of the phage and host bacterial strain used for its propagation should be used to interrogate for known therapeutically deleterious elements (e.g., antibiotic resistance elements, toxin genes, prophages, integrase genes, regulators of integrase genes, and integrase-like genomic elements) in their genomes. Sequenced bacterial propagation hosts that do not produce toxins or harbor antibiotic resistance genes are preferred. Absent a trusted bacterial propagation host, the patient isolate itself may be considered a phage propagation host. In such cases, the isolate could be sequenced to show that it does not harbor toxin genes or antibiotic resistance genes, although given that phage therapy is often targeted at drug-resistant bacteria, this may not be possible. If there is no time to sequence the patient isolate’s genome, testing for specific toxins characteristic of the species (e.g., Shiga toxin for E. coli, toxic shock syndrome toxin 1 for S. aureus) may be considered, but it should be noted that bacterial species may encode toxins traditionally harbored in other species because of genetic exchange.

Phages may mediate transfer of genes to bacteria by lysogeny (or generalized transduction). Lysogeny (also known as lysogenic conversion) involves integration of a temperate phage genome into a bacterium’s genome. Phages that undergo lysogenic conversion are not ideal for therapeutic use, as they can render bacteria more virulent if the phage harbors deleterious genes. The ability of phages to undergo lysogeny may be assessed phenotypically and genotypically, with neither method being perfect. Genotypic methods involve sequencing phage DNA and analyzing sequence data for genomic elements involved in lysogeny (i.e., integrases, enzymes that mediate incorporation of phage DNA into bacterial DNA; integrase-like genomic elements; and regulators of lytic genes, such as repressor genes that help maintain prophages in a quiescent state). Phage genomes can also be examined for homology to known temperate phages, which suggests a lysogenic lifestyle. For example, a bioinformatics tool for computational evaluation of phage lifestyle such as the Phage Classification Tool Set (PHACTS) can be used (139). Integrase genes are diverse, so relying on sequence similarity to published genomes may be insufficient to accurately predict phage lifestyle. The annotation tool PHASTER, for example, detected only half of 147 integrase genes in prophages from 49 Salmonella enterica isolates (140). In addition, integrase genes are not necessary for a lysogenic lifestyle, with some phages replicating as plasmids in the lysogen. Further, mutations in single amino acid residues may render integrases inactive (141); thus, functional capacity of an integrase cannot be definitively known unless it is identical to an experimentally confirmed annotation. PHACTS does not universally provide clear classifications, and some differences are explained by specialized host-phage interactions that govern lifestyle by way of interference of integrases or other repressor genes (142). Philipson et al. recently described a multiplexed bioinformatics workflow to produce a fully assembled phage genome and to demonstrate that phages appear to be lytic and to not encode therapeutically deleterious genes (143). They propose that generated phage genomes be considered “finished” when they contain a single consensus sequence representing 100% of the genome with all open reading frames identified and a lack of population diversity, indicating purity of the sequence and verified via deep sequence coverage. PhageTerm is an example of a tool for determining genomic termini and phage packaging strategy (144). Sequences are evaluated for toxins and resistance genes, host, and laboratory contamination.

For phenotypic testing, phage plaques may be visualized to assess turbidity, which suggests that lysogeny may have occurred. This method is neither perfectly sensitive nor specific; even if plaques are clear, lysogeny may have happened but not be noticeable by eye. A sterile pipette tip touched to the plaque may be streaked on a plate of appropriate medium; bacteria that grow may be lysogens. These colonies can be retested for resistance to the phage; if resistant, they can be confirmed as lysogens if phages are detected after spontaneous excision or following induction with bacterial stressors or mutagens (e.g., mitomycin C, UV radiation, carbadox, peroxide, temperature stress). Caution is needed, however, as spontaneous phage resistance coupled with imprecise technique carrying over phage particles from plaques could appear to be a lysogen by this approach. Alternately, the genome sequence of a putative lysogen could be examined for the presence of a prophage absent from the parent. If the ability of a particular phage being evaluated to undergo lysogeny varies with the host bacterium assessed, definitive prediction of absence of lysogeny may be impossible.

Generalized transduction is another mechanism by which phages can mediate genetic transfer as segments of host DNA become incorporated into phage capsids by chance during the assembly phase of replication. Generalized transduction is an undesirable potential outcome of phage therapy, although it is impossible to completely mitigate. When recombinant phages encounter subsequent hosts, DNA may be incorporated into the bacterial genome through homologous recombination, resulting in transfer of DNA from one bacterium to another. Because of the stochasticity of phage assembly, most chromosomal sequences are transduced with roughly equal frequency.

Regarding phage formulation, preparations should be labeled with information on phage identity, purity, strength, stock expiration date, and storage conditions (115). An issue with phage stability is the occurrence of mutations that can impair viral fitness in phage stocks stored for long periods or accumulated during manufacturing and phage production. Development of manufacturing processes that minimize mutation of phage genomes is needed. Phage preparations should also be free of viable bacteria and fungi and contain minimal amounts of bacterial secreted products/debris (e.g., spores, endotoxin, exotoxins). Due to their classification as biological therapeutics, phage products used in the United States need to be manufactured under current good manufacturing practice (GMP) and adhere to USP requirements based on the type of application before administration. FDA guidance for GMP for phase I investigational products is available (145). Endotoxin levels in phage products need to be below acceptable limits set by the FDA and vary based on the route of administration. Methods to quantify endotoxin have been described (146); endotoxin requirements are <0.5 endotoxin units (EU)/ml for subcutaneous injections, <5 EU/kg of body weight/h for i.v. injections, and <0.2 EU/kg for intrathecal injections (147, 148). In industry and molecular biology applications, phage purification has often been carried out using polyethylene glycol precipitation followed by serial chloroform extractions, ultracentrifugation on a cesium chloride (CsCl) gradient, and subsequent dialysis to remove CsCl. Chloroform concentrations are regulated in medical products; therefore, the use of chloroform necessitates analysis of residual solvent concentration. CsCl is a theoretical safety risk (149), although residual CsCl concentrations in phage products would be unlikely to be significant (150). Other methods have been described to deplete endotoxin from phage preparations, such as polyethylene glycol, ultrafiltration, ultracentrifugation, gel filtration, anion-exchange chromatography, octanol extraction, deoxycholate extraction, endotoxin removal columns, and combinations thereof (151–156); not all work equally well for all bacterial species. Phage purification studies to date have mostly focused on phage yield and endotoxin removal, with little focus on removal of other bacterial toxins during phage purification. Gram-positive bacteria do not have endotoxin, but some may have other intrinsic toxigenic material, such as cell wall teichoic acid, which can be immunostimulatory.

Many pathogenic bacteria excrete toxins into their environment (e.g., S. aureus enterotoxins); although there are no strict limits on concentrations of staphylococcal enterotoxins in medical products, such toxins could be present at clinically relevant concentrations in phage products. A strategy to avoid toxins produced by pathogenic bacteria would be to propagate phages in nonpathogenic species if possible (e.g., propagation of phages targeted at S. aureus using Staphylococcus xylosus [157], although this potentially reduces the number of available therapeutic phages, as not all phages possess such broad-range activity). Another approach is to assess phage preparation effects on viability of a eukaryotic cell line, although how this might be used to include or exclude a particular preparation is unclear (158).

### Gaps in knowledge.

Computational tools to definitively exclude lysogeny based on phage genome sequence analysis require further development, as do criteria for defining the safety of phage formulations beyond endotoxin assessment.

## QUESTION 11: UNDER WHICH CONDITIONS SHOULD PHAGE BE QUANTIFIED IN CLINICAL SPECIMENS AND WHICH PARAMETERS MIGHT BE IMPORTANT FEATURES OF ASSAYS TO QUANTIFY PHAGE IN CLINICAL SPECIMENS?

### Suggestion.

Given the uncertainty as to the number of phages required at the infection site for maximal effect, the ARLG Phage Taskforce was unable to make a recommendation as to the circumstances under which phage concentrations should be measured in clinical samples. At this time, the ARLG Phage Taskforce suggests that determination of phage concentrations at sites of infection be limited to animal and clinical research studies and that such studies seek to determine the amount of phage required at the infection site for ideal effects. The ARLG Phage Taskforce was also unable to recommend a standard method for phage enumeration in clinical specimens; absent a standard method, considerations for attempting to quantify phages in clinical specimens are addressed below.

### Rationale.

As with antibiotics, there may be a need for accurate and reproducible methods that detect and quantify phages in complex clinical samples obtained from infection sites (84). Such data may be helpful to determine optimal dose, route of administration, dosing frequency, and treatment duration. However, few studies have assessed phage concentrations at infection sites. Ideally, assays that quantify phages in clinical specimens should be designed such that naturally present (nontherapeutic) phages are not detected.

The double agar overlay method and spot modification thereof (Question 7) may be reconfigured to quantitative assays to determine phage titers by using serial dilutions of known concentrations of phage as a comparator to unknown amounts of phage, with phage concentrations expressed as PFU per milliliter of the assayed preparation. This may underrepresent the number of active phages if a single plaque reflects activity of multiple phage particles, which, though theoretically possible, is likely rare. Likewise, liquid-based methods (Question 7) can be reconfigured to quantitative assays. Quantitative (including digital droplet) PCR can be used to enumerate phages based on detection of phage nucleic acid (84). In most cases, phage quantitative PCR platforms use probes for specificity (91, 159). Quantitative PCR may be sensitive and reproducible and, while accurate and potentially configurable as a high-throughput test, may be expensive and technically challenging. Upfront equipment costs, sample nucleic acid extraction, the need for specific primer and, if used, probe design for each phage species (or related phages) based on *a priori* sequencing results, and assay validation are among the complexities. Finally, quantitative PCR-based viral quantitation cannot discriminate between viable and nonviable phages and may therefore overestimate functional phage concentrations.

The ARLG Phage Taskforce was unable to recommend a standard method for phage enumeration in clinical specimens but favored the double agar overlay method as it measures viable phages, which is the primary interest with use of lytic phages. However, the members of the ARLG Phage Taskforce suggest that it is reasonable to perform both double-agar overlay and quantitative PCR on the same clinical specimen for enumeration of phage where possible, given that there is no clear gold standard. If a rapid high-throughput assay is needed to quantify a specific phage in multiple samples, the ARLG Phage Taskforce suggests that it may be possible to use quantitative PCR with the addition of a double-agar overlay method correction coefficient (if shown to be accurate) to translate results to viable phage counts. Even if correlations can be established, the ARLG Phage Taskforce suggests that it is prudent to continue to use the double-agar overlay method alongside quantitative PCR until the relationship between results of the two approaches and reproducibility of testing are established with certainty for the phage under investigation.

In addition to methods to quantify phage preliminarily introduced in Question 7 and above, phage particles have been enumerated in other ways. Transmission electron microscopy (TEM) after negative staining and epifluorescence microscopy after staining with DNA fluorochromes have been used to enumerate phage but are labor-intensive, time-consuming, and expensive. TEM is impractical for processing many samples concurrently and cannot be used for complex clinical samples (84, 160). Flow cytometry and nanoparticle detection by laser-illuminated optical microscopy, mass spectrometry, and next-generation sequencing also have been used but are likely unsuitable for routine use (84, 91, 161, 162).

New detection and enumeration methods should address diverse specimen types, ranging from blood, tissue, synovial fluid, and urine to cerebrospinal fluid; have a suitable turnaround time; be easy to use in clinical laboratories; address the myriad of phages likely to be used, and have an ability to detect more than one phage in the same sample (relevant to cocktail administration). Finally, the clinical utility of such assays would need to be demonstrated; further animal and clinical research studies are needed. To this end, there are no existing clinical data that demonstrate phage concentrations that predict optimal net bacterial killing at an infection site, with clinical outcome potentially dependent on many factors, including, but not limited to, administration route, bacterial burden, phage density, bacterial phage susceptibility, bacterial doubling time, presence of biofilm, efficiency of phage adsorption and infection, latency period (i.e., time required for phage replication), burst size (i.e., number of phages released from a single lysed bacterial cell), and phage removal rates (163–166) (see Question 13).

If there is a critical need to determine phage concentrations in a specimen for clinical purposes, the ARLG Phage Taskforce suggests only measuring phage concentrations in patients with active infections, given the potential for self-amplification in the presence of bacteria. The ARLG Phage Taskforce was unable to identify the optimal time for phage enumeration postadministration but suggests a lag time between phage administration and quantification of at least a day to allow for self-amplification.

### Gaps in knowledge.

The clinical utility of bioassays or molecular detection methods for enumerating phages in clinical samples is not yet clear, and there is no understanding as to how well these methods correlate with one another or how they should be used in clinical practice. There is a need to better understand phage concentrations associated with optimal net bacterial killing *in vitro* and *in vivo*, not unlike the relationship between antibiotic concentrations and bacterial killing. To facilitate comparison across available quantification methods, standardized methods are needed; correlation between bioassays and molecular detection methods needs to be established. Advances in phage basic science, especially phage metagenomics, may facilitate identification of conserved genomic features as candidates for broad-range quantitative PCR detection assays that detect multiple different phages.

## QUESTION 12: WHAT TYPES OF PHAGE PRODUCTS ARE AVAILABLE, AND WHICH ARE PREFERRED FOR TREATMENT OF PATIENTS WITH ACUTE AND CHRONIC BACTERIAL INFECTIONS?

### Suggestion.

Phage have been used as monophage and in cocktails, prebiomanufactured (defined here as biomanufactured in advance), or biomanufactured in real time based on patient need (defined here as biomanufactured on demand). In addition, both natural and bioengineered phages have been used. Clinical data defining the optimal phage product type(s) are lacking. Like the situation for antibiotics, the ARLG Phage Taskforce endorses the use of a phage product that has been shown to have microbiologic activity against the targeted bacterial pathogen(s) and encourages clinicians to send the bacterial pathogen(s) to a phage testing center to identify specific phage(s) for a given patient isolate for development of an individualized phage product (with the caveats noted in Questions 6 and 7).

### Rationale.

In clinical practice, lytic phages may be administered as a cocktail or a single phage (54). Cocktails may be preferred to maximize the number of phages that target a specific bacterium and/or to broaden the spectrum of bacterial activity (i.e., target multiple bacteria in polymicrobial infections). Cocktails are also a strategy to optimize bacterial killing over time and minimize the potential for resistance emergence, as component phages may target different bacterial receptors with independent mutations in each receptor required to achieve resistance (26, 163, 167, 168). However, the theoretical benefits of this approach to maximize net bacterial killing and minimize resistance development have not been substantiated and require clinical validation. A drawback with cocktails is that individual phages usually require a decrease in concentration when mixed into a single dose. There is also the potential that component phages agglomerate (169) or interfere with one another by competing for the same bacterial receptor or drive cross-resistance (51, 169–172). Phages can be given sequentially, potentially avoiding antagonism among phages or driving of cross-resistance (51). Phages are often concurrently used with antibiotics, so antibiotic effects on phage therapy bear consideration.

Two primary paradigms exist to produce phage therapeutics: prebiomanufactured and on-demand biomanufactured based on individual patient need (173, 174). Prebiomanufactured phage products are prepared in advance and are available off the shelf for administration. Prebiomanufactured phage products are used for either empirical use (as discussed, empirical treatment is generally discouraged for compassionate use cases) or personalized use. Prebiomanufactured phage preparations can be personalized by selecting the most appropriate phages based on PST among an array of already-made phages (see limitations in Questions 6 and 7). Prebiomanufactured products may be comprised of one or many phages, with selection of phages based on the combined susceptibility of phages against a panel of representative clinical isolates, including multidrug-resistant isolates. In the United States, prebiomanufactured phage products are manufactured under current GMP regulations (175) and meet defined quality and uniformity specifications (see Question 10) (176).

It is possible to biomanufacture personalized phage products on demand; these are typically comprised of one or more phages with confirmed activity against an individual patient’s isolate(s) (54). With on-demand biomanufactured phage products, there is the potential to modify the phage product based on clinical response and/or emergence of phage resistance. The major drawback of on-demand biomanufactured phage products is the extended (i.e., weeks to months) associated manufacturing and regulatory timelines (177).

The Magistral Phage system is a regulatory framework developed in Belgium in which on-demand phages are prepared for individual patients in local compounding pharmacies (174). Typically, a pharmacist with a physician's prescription prepares such phage products by selecting phages from phage banks and incorporating them into a designated administration vehicle (174, 178). This decentralized strategy allows for nimble manufacturing of individualized therapies for patients as they need them (typically within hours to days), with no case-by-case approval required. There are some considerations with the use of magistral or compounded phage products. Phage selection criteria from a phage bank and appropriate library sizes have yet to be identified for compounded products (178). Preparations of magistral or compounded phage products do not have to necessarily adhere to GMP production requirements, as regulations for clinical production of phages have not been established in some Western countries (179).

### Gaps in knowledge.

Optimal phage products for empirical and personalized use against groups of common bacterial pathogen(s) should be developed and evaluated, preferably in well-designed, controlled clinical trials. Clinical studies are required to determine if single phage versus cocktails should be used. As with antibiotics, there likely is not one best practice for all clinical settings and infection types. To minimize production time of on-demand biomanufactured phages, there is a need to develop rapid screening methods to identify optimal phage(s) against patients’ bacterial pathogens (see Questions 6 and 7 for limitations of PST).

## QUESTION 13: WHAT ARE THE KEY PHARMACOKINETIC AND PHARMACODYNAMIC CONSIDERATIONS WITH SELECTING INITIAL PHAGE DOSES?

### Recommendation.

The ARLG Phage Taskforce suggests using the highest safe and tolerated dose of a phage product with endotoxin levels below the acceptable limits set by the FDA to maximize phage concentrations at the site of infection and infect as many host cells as possible with the first dose. Nevertheless, clinical outcomes are not always improved with higher relative to lower doses, reflecting the complexity of effective phage dosing. Phages with high microbiological susceptibility, high adsorption rates, large burst sizes, and short latency periods should be selected, where possible. To ensure adequate concentrations at the infection site, members of the ARLG Phage Taskforce favor repeated dosing over single-dose therapy based on currently available data. The ARLG Phage Taskforce favors relying on single-dose therapy only if repeated dosing is impractical (e.g., intraoperatively), in which case phages should be administered as a high-titer preparation directly to the infection site to maximize delivery to the infected site (see Question 11), and the possibility of adsorption to bacteria and self-amplification.

### Rationale.

Phages possess pharmacokinetic properties distinct from small-molecule antibiotics due to their large size, protein content, and self-replicating nature (180). Such size constraints may limit phage concentrations that can be attained per unit volume as well as phage uptake and transport across and into tissues *in situ*. Their protein-rich composition results in rapid elimination by the mononuclear phagocytic system and potential neutralization by antiphage antibodies (181, 182). Thus, phage concentrations at sites of infection, especially when systemically administered, are assumed to be substantially lower than the initial dose for most phage treatments before phage replication.

Preclinical and mathematical modeling studies suggest that factors responsible for net bacterial killing at the infection site include (i) density of bacterial cells (susceptible and resistant populations) and (ii) their growth rates, (iii) numbers of phages present, (iv) adsorption or infectivity rate of phages, (v) latency periods, and (vi) burst sizes and phage killing/removal/inactivation rates at sites of infection (163–166). A special feature of phage therapy is the potential for low-dose phage treatments, as phages are biologically replicating therapeutic agents. In contrast to antibiotics, small numbers of phages may result in profound increases in phage densities at infection sites “if the target population of bacteria is sufficiently dense and physiologically, and genetically, amenable to phage replication” (163). Based on data generated from preclinical infection models, it has been proposed that target bacterial cell densities need to be over a replication or proliferation threshold of 10^4^ CFU/ml for self-amplification to occur (165, 183).

Numbers of phages at sites of infection achieved by self-amplification or passive treatment required for optimal net bacterial killing and phage resistance prevention have not been established. Currently, the actual multiplicity of infection (MOI_actual_) ratio, which is the ratio of adsorbed phages to targeted bacteria, is the expression used to reflect the desired or targeted number of phages at infection sites relative to bacterial burden (164, 165). The target MOI_actual_ has been proposed to be 10 (165), with some clinical data suggesting that failure to achieve an MOI_actual_ of ≥10 is associated with treatment failure (11, 13, 16). Importantly, clinical trials data supporting the importance of this MOI_actual_ target are unavailable. Recent modeling data indicate that the MOI_actual_ should result in phage densities that far exceed the inundation threshold, which is the minimum phage concentration above which the bacterial population declines (184). When phage concentrations are substantially above the inundation threshold (i.e., inundative densities), phages are expected to infect all susceptible host cells in a relatively short time and bacterial counts to decline exponentially. Regardless of bacterial densities, several investigators suggest that a reasonable phage density capable of maximizing net bacterial killing at the infection site in a timely manner is 10^8^ PFU/ml (164, 166, 170, 184, 185).

It is important to note that reported MOI_actual_ and inundative phage density targets are based on mathematical modeling of preclinical infection model data (164–166, 184). Mathematical and simulation models are useful in quantifying conditions that optimize net bacterial killing by phages at infection sites, but their utility is restricted by validity of model assumptions and input parameter values (186). For example, ratios of viable to nonviable phage particles and numbers of absorbed phages to numbers of bacteria at infection sites are difficult to quantify and likely variable over a treatment course, making it difficult to estimate the MOI_actual_. Phages may bind bacterial debris from lysed bacteria and are heterogeneously distributed at infection sites, making it difficult to determine the actual number of active phages at an infection site that can infect bacterial cells (186, 187). Estimation of desired phage densities across modeling and simulation studies does not account for contributions of the immune system, bacterial host defense mechanisms [e.g., bacterial membrane vesicle production, which reduces phage virulence], concurrent receipt of antibiotics, and local host environments, all of which affect phage therapy outcomes (114, 185). Finally, modeling studies performed to support the development of phage therapy require clinical validation.

### Gaps in knowledge.

Studies examining the pharmacokinetic and pharmacodynamic properties of phages are scarce. Thus, standardization of pharmacokinetic and pharmacodynamic methodologies and their evaluations are required to enable informed clinical development of phages (164). Since phages require bacteria for self-amplification, further pharmacokinetic studies are needed to characterize phage abundance and concentration at common infection sites in infected patients with various dispositions (e.g., critically ill versus noncritically ill, immunocompromised versus immunocompetent, young versus old). Pharmacodynamic studies are needed to define optimal phage concentrations at infection sites to maximize net bacterial killing in infected patients. Studies are needed to determine whether the initial phage dose or phage replication most influences phage titers at infection sites (or whether both must be optimized) (170).

## QUESTION 14: WHAT ARE POTENTIAL ROUTES OF ADMINISTRATION FOR PHAGE THERAPY AND HOW SHOULD THEY BE SELECTED?

### Suggestion.

The best method for administering phage has not been established. Based on available data, the ARLG Phage Taskforce endorses i.v. phage administration for treatment of patients with infections that involve organs or systems in which phages have been shown to achieve titers/concentrate if benefits outweigh the risks. The ARLG Phage Taskforce also endorses direct administration when available data indicate the specific direct administration modality with a given phage product achieves viable phage titers at the intended infection site. While orally delivered phages hold promise, the ARLG Phage Taskforce suggests limiting their use to infections of the gastrointestinal tract or in combination with other routes of phage administration until more clinical data, especially with novel oral administration formulations, are available.

### Rationale.

The route of administration is likely to affect outcomes of treatment since efficacy is dependent on phage concentrations at the site of infection. Common routes of administration for patients with acute and chronic infections include systemic injection (e.g., i.v., i.p., intramuscular, and subcutaneous), oral, and direct administration (e.g., topical, intra-articular, intravesicular, inhalation, and bladder irrigation) (see Question 1) (173, 181). Use of phage for prevention and treatment of biofilm formation on medical devices has also been studied (188). Selection of administration routes for patients with acute and chronic infections has been largely empirical, with most published clinical data describing i.v., direct administration, or oral administration of phages (173).

Systemic injection routes are efficient ways to deliver phages and treat intravascular as well as a broad range of infections (181). Following systemic injection, phages are typically observed in the circulation shortly after administration. Due to rapid clearance from the bloodstream by the mononuclear phagocyte system, systemic phage concentrations decline exponentially within 8 to 12 h (189). Phages are also subject to inactivation by the complement system and circulating neutralizing antibodies (see Question 9) (181, 182). If phage therapy occurs over an extended period of time such that the generation of antiphage antibodies is possible, consideration should be given to adding or substituting a phage that does not cross-react serologically (185) (see Question 9). Generation of neutralizing antibodies appears to be most robust with the i.v. route of administration (182), which may be mitigated by specific dosing strategies (25). In addition to liver and spleen (part of the mononuclear phagocyte system), available data indicate that phages distribute into heart, skeletal muscles, bone marrow, thymus, kidneys, and bladder after systemic delivery and that their ability to concentrate in certain organs/systems is dose dependent. The lungs and brain are other organs where phages have been detected; “backwards” penetration of phages from the bloodstream to the gastrointestinal tract has also been demonstrated in animal studies (181).

Direct (e.g., intravesicular, intra-articular) or topical administration has been used to treat patients with PJI, VAD infection, osteomyelitis, and other infections (see Question 1). With systemic injections, only a fraction of the phage dose administered reaches nonvascular infection sites due to dilution, translocation inefficiencies, and phage loss or inactivation (190). In contrast, direct administration to infection sites may ensure a high titer of phages at the intended target site; however, results of direct administration have been mixed and best practices have not been established. One challenge with direct administration is that phages are nonmotile, proteinaceous particles that follow Bowman collision dynamics and have reduced efficacy when the selected route of administration does not facilitate timely and sustained distribution within spatially structured bacterial populations at the infection site (191). Another consideration is that components of direct administration applications may inactivate phages, limiting clinical utility (192).

Inhaled administration, a form of direct administration, has been used clinically for treatment of patients with respiratory tract infections, but the ability of inhaled phages to concentrate in anatomic components of the respiratory tract (e.g., bronchi, bronchioles, and alveoli) has not yet been systematically characterized in humans. In animal studies, phage titers achieved in the respiratory tract vary based on delivery method (e.g., inhaler, nebulization via jet, ultrasonic or vibrating mesh, aerosolization) (193–195), device, formulation (e.g., liquid, freeze-drying powder, spray-drying powder, aerosol), and phage (194). When delivering phages via aerosolization, care must be taken to ensure that the aerosolization method chosen does not reduce phage viability (193–195). Nevertheless, inhaled phage delivery, so long as active phages reach targeted bacteria at the infection site, may be an effective route of administration. Systems that aim to circumvent delivery challenges (e.g., controlled-release formulations) are under development (169, 171, 172).

Topical delivery has also been explored to deliver systemic titers of phages (196). Compared to systemic injection, direct or topical administration results in lower systemic concentrations, especially when applied to intact skin (181, 197). Further data with novel transdermal delivery systems are needed to determine future clinical utility.

Lastly, the oral route of administration has been long studied as a means for delivering phages both locally and systemically and is attractive due to convenience (181). Transcytosis, the transport of molecules from one side of a cell to the other via endocytosis and exocytosis, has been proposed as a mechanism for systemic absorption from gut epithelial cells (198). However, degrees of survival in the human gastrointestinal tract and systemic absorption are uncertain, and oral administration is a less efficient means for achieving systemic therapeutic phage concentrations relative to other administration routes. Factors that may affect passage through the gastrointestinal tract include acidity of the stomach, host immune responses, and inhibitory effects of intestinal mucins (95, 181). Recovery of phages in feces can be used as a marker of survival in the gastrointestinal tract. Strategies to enhance stability of phages at unfavorable pH or temperatures and avoid phage elimination from the body to maintain active phages at infective doses are being pursued (e.g., encapsulation of phages in various matrices) (199). To date, phages have been detected in blood, urine, and feces in a dose-dependent manner in animal models following oral phage administration (200, 201). However, recovery of phages was inconsistent and, in some cases, not detectable in blood (202).

### Gaps in knowledge.

Additional human and animal studies are needed to characterize the distribution of phages in the central and peripheral tissues/organs/systems/compartments of interest (i.e., common sites of infection) in infected patients after injection and oral administration. Data on the ability of specific direct administration modalities to achieve therapeutic phage titers at infection sites are required. Oral administration systems that optimize systemic phage delivery would be ideal. Additional studies will be critical to inform dosing in terms of phage concentration, frequency, and duration of treatment. Given the diversity of phages and their bacterial hosts, quantitative pharmacokinetic and pharmacodynamic assessments will ideally need to be completed for each phage product, each host (the patient's bacterial pathogen), and each patient.

## QUESTION 15: WHAT DOSING FREQUENCY AND DURATION FOR PHAGE THERAPY SHOULD BE USED?

### Suggestion.

There are insufficient data for the ARLG Phage Taskforce to make definitive suggestions on the optimal dosing frequency and duration of phage therapy for any route of administration or any specific infection type. Available data suggest that phages need to be redosed to maximize phage concentrations at sites of infection, but ideal frequencies and durations of administration are unclear. The literature regarding the clinical use of phage therapy has not indicated clear safety concerns, supporting the use of repeated dosing for extended durations, especially to maximize concentrations at infection sites. Members of the ARLG Phage Taskforce hypothesize that the required dosing frequency and duration may vary as a function of several factors, including the phage product, pathogen, disease burden, and location of the infection. Until more data are available, members of the ARLG Phage Taskforce suggest that patient responses inform durations of therapy.

### Rationale.

Phage dosing, irrespective of route of administration, thus far has been largely empirical. In most published cases, phage formulations have been administered daily (8, 9, 21, 22, 24, 34, 45, 50), twice-daily (5, 8, 23, 25, 27, 35, 44, 47–50, 52, 58, 62, 138), every 6 to 8 h (7, 20, 32, 36, 40, 46, 48, 54, 55, 57), or by extended infusion over hours (49) (Table 1). Iredell et al. detected phage DNA in blood up to 12 h after dosing with staphylococcal phages in humans with bloodstream infections (56). This served, in part, as the rationale for 12-h dosing. Many patients are treated via more than one route of administration (e.g., i.v. and direct application) (5, 7, 44, 45, 49, 57, 58, 62, 138) (Table 1). Durations of therapy have spanned from 1 day (17, 37, 38, 41–43, 50, 51, 64) to months (7, 8, 20, 23, 25, 34, 54, 62, 173) (Table 1). Limited studies in humans and animals show recovery of phages from feces and blood after oral administration to be dose and duration dependent (181). *In vitro* and animal direct administration studies have shown a spectrum of efficacy based on dose and duration (107, 203–213). Independent of administration route, resistance to phages is more likely to occur with repeated dosing and prolonged treatment courses (14, 214, 215). While phage resistance emergence is undesirable, there are limited clinical data indicating that in some scenarios, bacteria may become more susceptible to antibiotics and less virulent when they develop phage resistance (9, 51, 54) (see Question 2).

### Gaps in knowledge.

Substantial dosing-related knowledge gaps exist for all routes of phage administration. Validated preclinical infection pharmacokinetic/pharmacodynamic models and controlled clinical trials are necessary to determine optimal phage dosing regimens and durations of therapy for patients with acute and chronic bacterial infections. Through such studies, optimal phage therapy practices can be established. As part of these studies, it will be important to determine when phages should be administered with respect to dosing specific antibiotics.

## CONCLUSIONS

Phage therapy has reemerged as a potential treatment for refractory infections in recent decades due to the increasing need for alternative or adjunctive anti-infectives to conventional antibiotics. Several uncontrolled case studies report successful clinical outcomes. On the other hand, clinical failures are likely underreported, and the few randomized controlled trials that have been conducted have failed to show benefit. Thus, no recommendation can be made to support routine clinical use of phage therapy under any circumstance; much is unknown about the efficacy of phage therapy and potential reasons for failure, such as dosing, frequency of dosing, duration of therapy, routes of administration, interactions with antibiotics, interactions with other phages, emergence of phage resistance, inadequate phage delivery, and superhost immune response, to name a few. While more clinical research studies are needed, this document is intended to identify the key questions for clinicians to address when considering phage therapy for individual experimental clinical use. The wide spectrum of potential clinical indications, the safety and tolerability of phages, the requirements for safe administration of phage therapy, current regulatory pathways for expanded access, and a lack of (and need for) standardized assays for PST and phage quantification methods are highlighted. The importance of individualized therapy and confirmation of lytic activity before treatment, whenever possible, are highlighted, recognizing a need to develop standardized, accurate methods for the latter. Synergies that may occur when antibiotics are used with phage therapy are underscored. Although phage therapy is currently reserved as salvage therapy, ultimately the hope is that phages, if shown to be beneficial, will be able to be utilized earlier during infection, perhaps reducing up-front use of antibiotics, helping to preserve them. By no means comprehensive or final, it is hoped that this document can lay the groundwork for rational phage selection and dosing as more research is done to enhance understanding of the complexities of the phage-bacterium-human interplay.
